# Methylation and transcriptomic expression profiles of HUVEC in the oxygen and glucose deprivation model and its clinical implications in AMI patients

**DOI:** 10.3389/fgene.2023.1293393

**Published:** 2023-12-07

**Authors:** Yuning Tang, Yongxiang Wang, Shengxiang Wang, Runqing Wang, Jin Xu, Yu Peng, Liqiong Ding, Jing Zhao, Gang Zhou, Shougang Sun, Zheng Zhang

**Affiliations:** ^1^ The First School of Clinical Medicine, Lanzhou University, Lanzhou, China; ^2^ Gansu Key Laboratory of Cardiovascular Diseases, The First Hospital of Lanzhou University, Lanzhou, China; ^3^ Department of Cardiology, Lanzhou University Second Hospital, Lanzhou, China; ^4^ Cardiovascular Clinical Research Center of Gansu Province, Lanzhou, China; ^5^ Heart Center, The First Hospital of Lanzhou University, Lanzhou, China; ^6^ School of Life and Environmental Sciences, Minzu University of China, Beijing, China

**Keywords:** DNA methylation, oxygen-glucose deprivation, acute myocardial infarction, mRNA sequence, brain-derived neurotrophic factor, tumor necrosis factor superfamily member 10

## Abstract

The obstructed coronary artery undergoes a series of pathological changes due to ischemic-hypoxic shocks during acute myocardial infarction (AMI). However, the altered DNA methylation levels in endothelial cells under these conditions and their implication for the etiopathology of AMI have not been investigated in detail. This study aimed to explore the relationship between DNA methylation and pathologically altered gene expression profile in human umbilical vein endothelial cells (HUVECs) subjected to oxygen-glucose deprivation (OGD), and its clinical implications in AMI patients. The Illumina Infinium MethylationEPIC BeadChip assay was used to explore the genome-wide DNA methylation profile using the Novaseq6000 platform for mRNA sequencing in 3 pairs of HUVEC-OGD and control samples. GO and KEGG pathway enrichment analyses, as well as correlation, causal inference test (CIT), and protein-protein interaction (PPI) analyses identified 22 hub genes that were validated by MethylTarget sequencing as well as qRT-PCR. ELISA was used to detect four target molecules associated with the progression of AMI. A total of 2,524 differentially expressed genes (DEGs) and 22,148 differentially methylated positions (DMPs) corresponding to 6,642 differentially methylated genes (DMGs) were screened (|Δβ|>0.1 and detection *p* < 0.05). After GO, KEGG, correlation, CIT, and PPI analyses, 441 genes were filtered. qRT-PCR confirmed the overexpression of VEGFA, CCL2, TSP-1, SQSTM1, BCL2L11, and TIMP3 genes, and downregulation of MYC, CD44, BDNF, GNAQ, RUNX1, ETS1, NGFR, MME, SEMA6A, GNAI1, IFIT1, and MEIS1. DNA fragments BDNF_1_ (r = 0.931, *p* < 0.0001) and SQSTM1_2_NEW (r = 0.758, *p* = 0.0043) were positively correlated with the expressions of corresponding genes, and MYC_1_ (r = −0.8245, *p* = 0.001) was negatively correlated. Furthermore, ELISA confirmed TNFSF10 and BDNF were elevated in the peripheral blood of AMI patients (*p* = 0.0284 and *p* = 0.0142, respectively). Combined sequencing from *in vitro* cellular assays with clinical samples, aiming to establish the potential causal chain of the causal factor (DNA methylation) - mediator (mRNA)—cell outcome (endothelial cell ischemic-hypoxic injury)-clinical outcome (AMI), our study identified promising OGD-specific genes, which provided a solid basis for screening fundamental diagnostic and prognostic biomarkers of coronary endothelial cell injury of AMI. Moreover, it furnished the first evidence that during ischemia and hypoxia, the expression of BNDF was regulated by DNA methylation in endothelial cells and elevated in peripheral blood.

## 1 Introduction

Acute myocardial infarction (AMI) is a common but critical disease with high mortality and morbidity rates. Once the plaque in the coronary artery ruptures and the blood flow is interrupted, it not only leads to injury in cardiomyocytes due to reduced oxygen and glucose supply, but also triggers a series of pathophysiological changes in endothelial cells. Recently, studies have illustrated that endothelial cells release an enormous number of extracellular vesicles following an AMI event, including exosomes and microvesicles, which facilitate myocardial repair and angiogenesis (Loyer et al., 2018; Liu et al., 2020). Moreover, endothelial cells play an essential role in cardiac ischemia/reperfusion (I/R) injury (Cochain et al., 2013) and the revascularization of the surrounding vessels in the infarcted area (Li et al., 2019). Although early reperfusion after AMI could be beneficial to limit the infarct size, delayed diagnosis of patients with massive myocardial injuries often leads to poor outcomes, suggesting that new treatment modalities are urgently required to promote myocardial perfusion, cardiac repair, and regeneration for this subset of high-risk AMI patients. Through revascularization and the promotion of angiogenesis, the apoptosis and necrosis of cardiomyocytes can be effectively reduced, which is the key treatment for myocardial infarction to save cardiomyocytes (Virani et al., 2020). Therefore, clarifying the underlying pathomechanisms of endothelial cell injury during AMI is necessary, as it enables early diagnosis and improved treatments (Wu et al., 2015).

DNA hypermethylation-mediated suppression of gene expression is the primary type of epigenetic regulation in living organisms, including humans. DNA methylation occurs at the C5 position of cytosine by conjugation of methyl groups, most commonly known as 5′-C-phosphate-G-3' (CpG) dinucleotides (Razin et al., 1984). Hypermethylation of specific promoter regions of acute coronary syndrome (ACS)-associated genes can inhibit their transcriptional activation and alter their biological functions, leading to an increased risk in these patients (Lu et al., 2013). Besides, genome-wide and site-specific DNA methylation alterations have been found in cardiovascular disease (Afzali et al., 2013) and ACS (Li et al., 2010; Kim et al., 2015) patients and disease models. Previous studies suggest that modulation of DNA methylation might be a promising tool for early ACS prediction and diagnosis (Li et al., 2017; Soares et al., 2020; Long et al., 2021; Park et al., 2021; Schiano et al., 2022). However, there are no disease-oriented datasets of systematic analyses of AMI-linked changes in genomic DNA methylation status in endothelial cells. Oxygen-glucose deprivation (OGD)-induced hypoxia and metabolic stress in cultured endothelial cells is used to mimic the conditions experienced by coronary arteries during AMI (Zhang et al., 2021). Hence, in this study, we used the human umbilical vein endothelial cells (HUVEC) stressed with the OGD model to explore the DNA methylation patterns in endothelial cells and delineate their significance in acute ischemic-hypoxic conditions. In addition, we analyzed peripheral blood samples from AMI patients to investigate potential biomarkers released into the circulation during the onset of AMI and to evaluate their clinical significance.

## 2 Materials and methods

**FIGURE 1:**
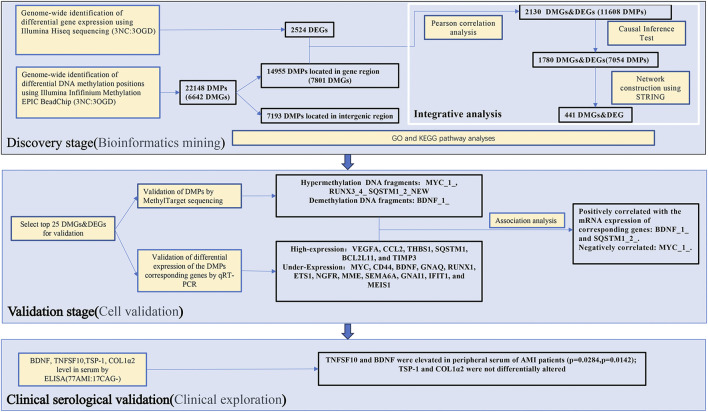
A flow chart illustration of the study design and experimental strategies.

The pramiry materials and methods used in the experiments were listed below, and the flow chart of the whole study was shown in Figure 1.

### 2.1 Study subjects

The present study recruited 71 AMI patients and 17 age-matched control subjects who were negatively diagnosed with coronary angiography (CAG) from the Heart Center of the First Hospital of Lanzhou University (Lanzhou, China) between June 2021 and January 2022. The patients with non-ST-elevation myocardial infarction (NSTEMI) were those with no characteristic ST-segment elevation on ECG but with chest pain symptoms and elevated levels of myocardial enzymes (TNI>0.023 ng/mL) for more than 4h, as well as confirmed vascular occlusion in the coronary angiography. In contrast the control subjects had no coronary stenosis confirmed by coronary angiography and had normal physical examinations and ECG findings. The patients with tumours, severe hepatic or renal abnormalities, severe infections, and any other conditions that the investigators deemed inappropriate for participation were excluded from this study. This study was approved by the Ethics Committee of the First Hospital of Lanzhou University (Approval No. LDYYLL-2023-42).

### 2.2 Blood sampling

Peripheral venous blood samples from the elbow vein were collected from the patients in EDTA-coated tubes before the coronary angiography. Tubes were turned upside down 8-10 times to mix the blood with EDTA and centrifuged at 3500 rpm for 10 min. The uppermost layer was aspirated as plasma and stored at −80°C for downstream analysis.

### 2.3 Cell culture and OGD treatments

The HUVEC line was purchased from ATCC (Manassas, VA, United States) and cultured in the endothelial cell medium (ECM, ScienalysisnCell, CA, United States) containing 5% fetal bovine serum, endothelial cell growth supplement, and 1X penicillin/streptomycin solution. Cells were passaged into a 1:3 ratio when the density reached 80%-90%. Cells between the third and eighth passages were taken for experiments. OGD is used as an *in vitro* model to mimic *in vivo* hypoxia-ischemia injury. Cells were plated, walled for 12h, and washed three times with PBS. Then the culture medium was replaced with glucose-free DMEM (Meilunbio, Dalian, China) followed by incubation in a chamber containing a mixture of 94%N_2_, 5% CO_2_ and 1% O_2_ (Thermo Fisher Scientific, MA, United States) for certain hours (Baldea et al., 2018). The normal control group was maintained all the time in a complete medium. Cells were collected immediately after the treatment and stored at −80°C for future use. All studies were performed using 3 biologically independent sets of experiments.

### 2.4 MTT assay

The HUVEC cells were incubated overnight to adhere to the wall. After treatment with OGD for 0h, 2h, 4h, 6h, and 8h, 10 μL of MTT solution (concentration of 5 mg/mL) (solarbio, Beijing, China) was added and incubated for 4 h. The culture solution was carefully aspirated. 150μL of dimethyl sulfoxide (solarbio, Beijing, China) was added to each well, and the wells were shaken horizontally for 10 min at low speed on a shaker. The absorbance of each well was measured at 490 nm on an enzyme marker (Tecan).

### 2.5 RNA isolation

According to the manufacturer’s instructions, the total RNA was isolated from HUVECs using the M5 universal RNA Mini Kit (Mei5 Biotechnology, Beijing, China). In brief, the culture medium was thoroughly aspirated from the treated HUVECs, and 350 μL of RLT lysis buffer was added. The cells were lysed by repeatedly blowing. An equal volume of 70% ethanol was added to the mixture and immediately mixed by blowing. The mixture was then transferred to an adsorption column (RA) placed in a collection tube, and centrifuged at 13,000 rpm for 30 s, with the flow-through being discarded. Next, 700 μL of proteinase-free buffer (RW1) was added, incubated at room temperature for 30 s, and centrifuged at 13,000 rpm for 30 s, followed by discarding the flow-through. Subsequently, 500 μL of wash buffer (RW) was added, centrifuged at 13,000 rpm for 30 s, and the flow-through was discarded. The wash step was repeated. The adsorption column (RA) was placed back into an empty collection tube, and centrifuged at 13,000 rpm for 2 min to remove excess wash buffer. The adsorption column (RA) was then transferred to a 1.5 mL centrifuge tube, and 30-50 μL of RNase-Free H2O was added to the middle portion of the adsorption membrane, depending on the expected RNA yield. The mixture was incubated at room temperature for 1 min, followed by centrifugation at 13,000 rpm for 1 min to obtain the RNA solution. The purity of RNA was assessed by a Nanodrop 2000 spectrophotometer (Thermo Fisher Scientific, Waltham, United States) and the concentration was measured on an Invitrogen Qubit 3.0 spectrophotometer (Thermo Fisher Scientific, Waltham, United States). RNA quality was evaluated on a Bioanalyzer 2,100 (Agilent Technologies, Santa Clara, United States), and DNA integrity was analyzed by agarose gel electrophoresis.

### 2.6 DNA methylation and expression profiling

The cells prepared in step 1.3 were collected into 1.5 mL centrifuge tubes, added 500 μL of proteinase K solution (10 mg/mL) to each tube. Extracted DNA according to the instructions from the nucleic acid purification kit (Concert, Xiamen, China) and performed quality assessment. Treated DNA with bisulfite, followed by whole genome amplification (WGA) and fragmentation to generate fragmented DNA. Precipitated and resuspended the obtained DNA then hybridized it with the beads on the chip, where specific bases were attached. Washed away the unhybridized and non-specifically hybridized DNA, and performed single-base extension reactions on the chip, incorporating detectable label moieties. Placed the processed chip into a scanner and used laser excitation to stimulate the fluorescence emitted by the single-base extension products on the chip. The scanner captured the fluorescence signals emitted by the fluorescent moieties. The DNA methylation level was measured using the Illumina Infinium Methylation EPIC v2.0 BeadChip and processed with ChAMP package in R (https://bioconductor.org/packages/release/bioc/html/ChAMP.html). The cDNA samples prepared in step 1.5 were sequenced for RNA expression profiling using the Illumina Novaseq 6,000 system (von Kanel and Huber, 2013; Bibikova and Fan, 2010). The raw reads were filtered using TrimGalore (http://www.bioinformatics.babraham.ac.uk/projects/trim_galore/), and the filtered data were analyzed using FastQC software. The methylation level at an individual locus was reported as b a value, which varied from 0 (unmethylated) to 1 (fully methylated).

Based on the defined differentially methylated positions (DMPs), hierarchical clustering was conducted using Cluster 3.0 and Java TreeView software. DMPs located in the gene region were assigned to the corresponding genes, which were defined as DMGs (Zhu et al., 2019).

The differentially expressed genes (DEGs) between the OGD and control groups were identified using Deseq2 software. The DEGs were selected by *p* < 0.05 and |log2 (fold change, FC)|>1, where log2(FC) > 1 and log2(FC)<-1 respectively indicated upregulated and downregulated genes. The combined datasets, including 3 OGDs and 3 negative controls (NCs), were normalized by the BMIQ algorithm.

The gene ontology (GO), Kyoto Encyclopedia of Genes and Genomes (KEGG), and disease ontology (DO) enrichment analyses were performed using the clusterProfiler. STRINGdb (Szklarczyk et al., 2017; Szklarczyk et al., 2019) was used to analyze DEGs’ protein-protein interaction (PPI) network.

### 2.7 Integrative analysis of DNA methylation and gene expression data

To determine whether the methylation level was associated with the expression profile of the concerned gene, a correlation analysis was performed by R as well as Causal Inference Test (CIT) Analysis by the R package Causallmpact (https://google.github.io/CausalImpact/). Correlation analysis screened out CpG loci whose methylation levels were negatively correlated with the unique DEGs expression (r > 0.8). The CpG locus is within 1,000 kb of the gene it regulates and therefore has a cis-regulatory effect on the gene. Subsequently, the screened CpGs and DEGs were analyzed by CIT to find out the differentially methylated CpG loci and DEGs that were statistically causally related. The STRING database was used for the PPI analysis of causally related DEGs and differentially methylated genes (DMGs). The fast greedy algorithm of the igraph package was used to cluster the constructed internetworks and partition it into different modules for plotting.

### 2.8 Quantitative real-time PCR (qRT-PCR) analysis

The extracted RNA was used to synthesize complementary DNA (cDNA) for the downstream qRT-PCR analysis using SYBR Green Master Mix (ABI/QuantStudioTM DX, United States). The primer sequences of each gene were listed in Table 1. The relative gene expression level was determined by the 2^−ΔΔCT^ method, using GAPDH as an internal reference.

**TABLE 1:** Primer sequence.

No.	Gene	Forward Sequence (5′-3′)	Reverse Sequence (5′-3′)
1	MYC	TCG​GAT​TCT​CTG​CTC​TCC​TCG	TCT​TCT​TGT​TCC​TCC​TCA​GAG​TCG
2	VEGFA	CTT​CAA​GCC​ATC​CTG​TGT​GCC	GTT​TGA​TCC​GCA​TAA​TCT​GCA​TGG
3	CD44	GTC​GCT​ACA​GCA​TCT​CTC​GG	CAG​AGC​TTT​CTC​CAT​CTG​GGC
4	BDNF	TGG​AGG​TGG​GGC​ATG​GTA​TT	AAA​GCA​CGA​GGT​CCA​AGC​AG
5	CCL2	TGA​AAG​TCT​CTG​CCG​CCC​TT	GGG​GCA​TTG​ATT​GCA​TCT​GGC
6	TSP-1	AAC​ACG​GAC​CCC​GGC​TAC​AA	TAC​GGG​GCT​TGC​ACA​CCT​GTT
7	TNFSF10	TGG​CTA​TGA​TGG​AGG​TCC​AGG​G	GAC​TGC​AGG​AGC​ACT​GTG​AAG​A
8	COL1A2	CCC​AGA​GTG​GAG​CAG​TGG​TTA	CCG​GAT​ACA​GGT​TTC​GCC​AG
9	GNAQ	AGA​GTT​CGA​GTC​CCC​ACC​AC	CCC​CCT​ACA​TCG​ACC​ATT​CTG​A
10	SQSTM1	GTA​GCG​TCT​GCG​AGG​GAA​AG	TGC​GAG​AAG​CCC​TCA​GAC​A
11	RUNX1	CCC​ATC​GCT​TTC​AAG​GTG​GT	TGG​CTG​CGG​TAG​CAT​TTC​TC
12	ETS1	CAG​ATG​CCG​ACG​AGT​GAT​GG	GAG​TCC​AAC​CAA​CAC​GGC​TG
13	NGFR	CAC​CGA​CAA​CCT​CAT​CCC​TGT	CTT​GCA​GCT​GTT​CCA​CCT​CTT​G
14	BCL2L11	ACC​AAA​TGG​CAA​AGC​AAC​CTT​C	GCT​CTG​TCT​GTA​GGG​AGG​TAG​G
15	LUM	GCA​GTG​TCA​AGA​CAG​TAA​GGA​TTC	ACC​ACC​AAT​CAA​TGC​CAG​GA
16	MME	CTG​GAG​ATC​AGC​CTC​TCG​GT	TCG​TAG​GTT​GCA​TAG​AGT​GCG
17	SEMA6A	CGT​TGC​ACT​GTT​TGC​AGA​TGG	TGA​ATC​GTG​CTT​GAC​GGT​CC
18	TIMP3	ACC​GAG​GCT​TCA​CCA​AGA​TG	CCA​TCA​TAG​ACG​CGA​CCT​GT
19	GNAI1	GGT​GCC​CTT​CTG​GGA​ACT​AC	GTC​CAA​TGC​TGG​AGG​ACT​CG
20	IFIT1	CGC​TGG​GTA​TGC​GAT​CTC​TG	CCT​GCC​TTA​GGG​GAA​GCA​AAG
21	LRP5	CAA​CGG​CAG​GAC​GTG​TAA​GG	CAC​GAT​GTC​GGT​GAA​GTC​CG
22	MEIS1	CTG​CAC​TCG​CAT​CAG​TAC​CC	GGG​AAG​AGG​GGG​TGT​CCA​TA

### 2.9 Targeted methylation sequencing

DNA extraction was performed after remodeling, and the samples were processed using the EZ DNA Methylation-Gold Kit (ZYMO, CA, United States) to convert unmethylated cytosine C) to uracil U). Multiplex PCR tests were conducted to target specific fragments of the samples, and specific label sequences were added. All samples’ Index PCR amplification products were mixed in equal amounts, and the MethylTarget sequencing library was obtained by gel purification. After accurately quantifying the molar concentration of the library, high-throughput sequencing was performed on the Illumina Hiseq/Miseq platform using a 2 × 150 bp/2 × 250 bp paired-end sequencing mode, resulting in FastQ data. The analysis methods were as shown in step 1.6.

### 2.10 The enzyme-linked immunosorbent assay (ELISA)

The ELISA kit (Elabscience, Wuhan, China) was used to detect extracted plasma samples, according to the manufacturer’s instructions.

### 2.11 Statistical analysis

Statistical analyses were performed using GraphPad Prism 7 (San Diego, CA, United States). D’Agostino-Pearson omnibus normality test was used to evaluate the normality of the distribution of the variables. Normally distributed values were expressed as the mean ± standard deviation (SD), and the differences between the groups were analyzed by unpaired *t*-test. Non-normally distributed data were expressed as median and quartile and analyzed by the Mann-Whitney test. A *p*-value of <0.05 was considered statistically significant.

## 3 Results

### 3.1 Identification of DEGs in the HUVEC-OGD group by mRNA sequencing

**FIGURE 2:**
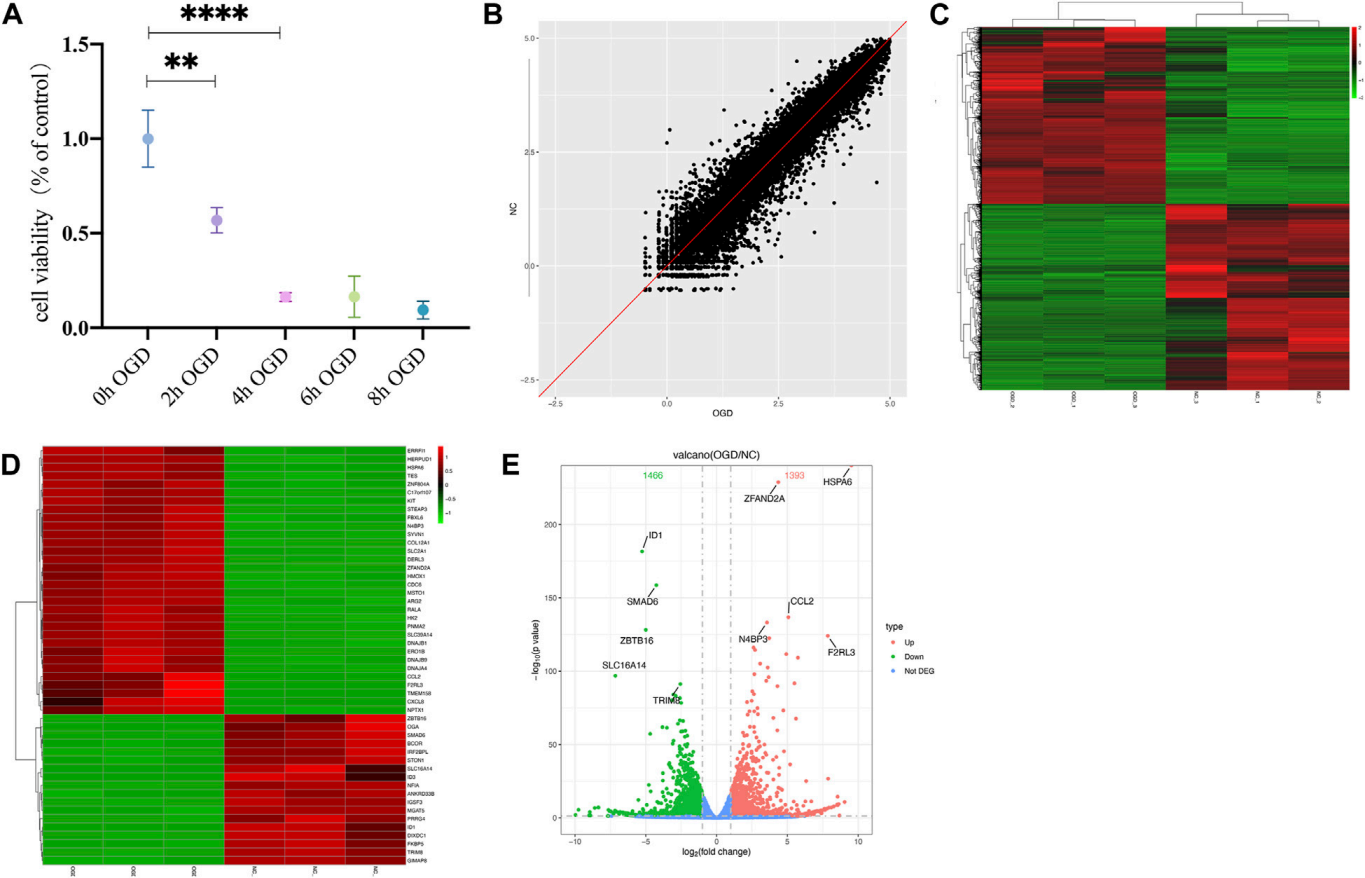
Differentially expressed genes (DEGs) were analyzed by a scatter plot. **(A)** MTT results of HUVECs at specific time points of OGD treatment. **p < 0.01 and ****p < 0.0001vs control group; At 2h, the cell viability decreased almost 50% and 20% at 4 h. **(B)** Each point in this plot represents the logarithmic value of the mean expression of a gene after correction in two groups-the *X*-axis for the OGD group and the *Y*-axis for the NC group. The points of the diagonal surface represent relatively highly expressed genes in the corresponding group. **(C)** Heat map of clustering of DEGs. The *X*-axis indicates different samples and *Y*-axis for different genes; the red color represents highly expressed and green for genes with decreased expressions. **(D)** Heat map of top 50 significant DEGs. **(E)** Volcano plot of DEGs. Red dots indicate genes with upregulated expressions in the OGD group relative to the NC group, green dots for downregulated, and blue dots for genes with no significant differences.

**FIGURE 3:**
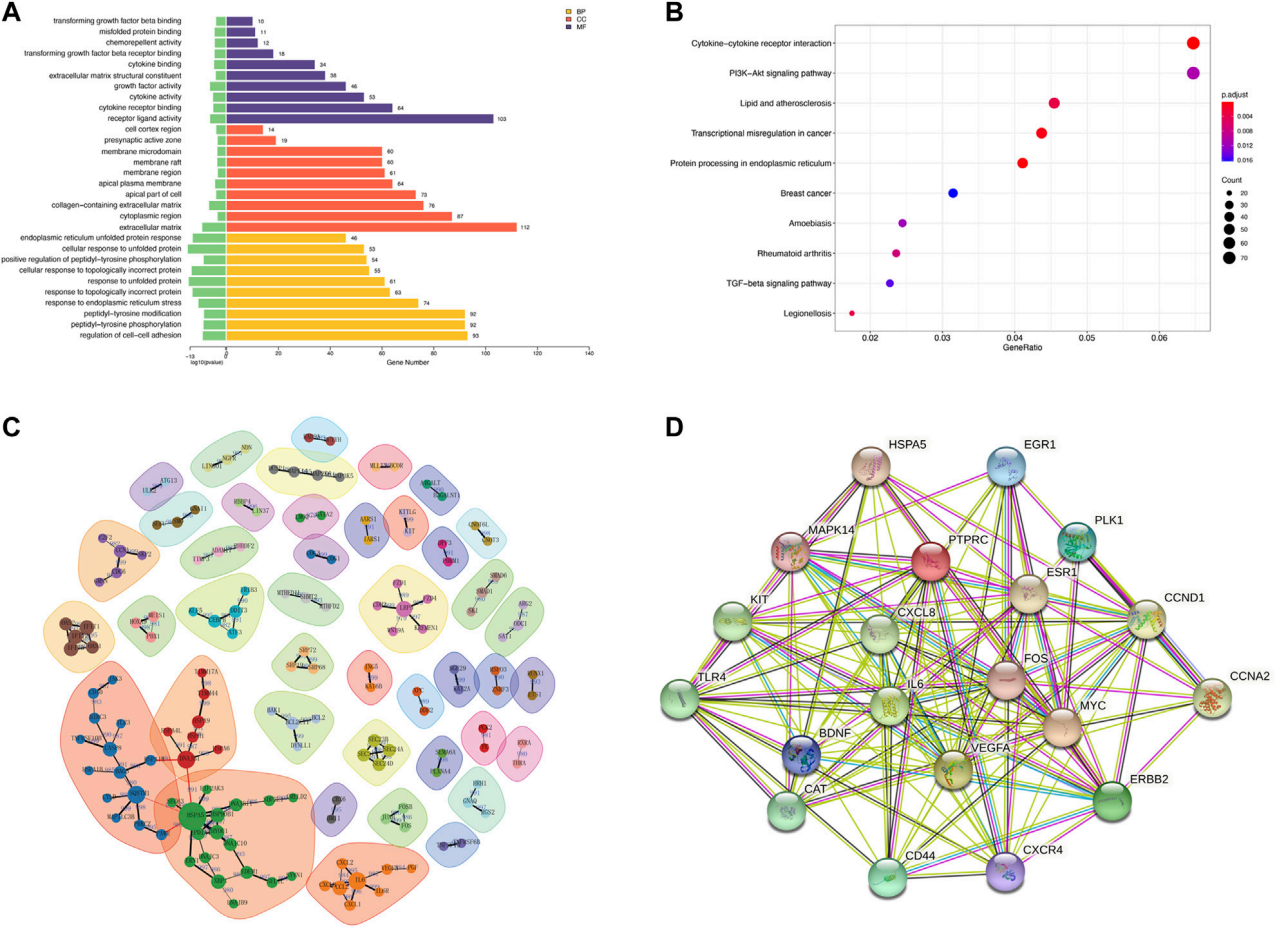
Functional enrichment and protein-protein interaction (PPI) analyses of DEGs. **(A)** Gene ontology (GO) enrichments in a bar graph. The log10 (*p* values) are shown in green at the left of the *X*-axis. The categories of BP, MF, and CC in GO analysis were illustrated in yellow, purple, and red, respectively. **(B)** The KEGG scatter plot. The horizontal coordinate represents the GeneRatio and the vertical coordinate is the -log10 (Q-value). **(C)** PPI analysis of top 1,000 DEGs. The nodes in the graph are proteins, and the edges are reciprocal relationships. Different clusters of modules are indicated by different base colors. **(D)** PPI analysis of the top 20 hub genes.

The MTT results showed a significant decrease in cellular activities of HUVECs at 4 h of OGD treatment (Figure 2A). Therefore, we used HUVECs sequencing at the 4 h time point of OGD induction to identify disease-associated deregulated gene expressions and the involved signaling pathways. About 2,524 DEGs were detected by examining mRNA expressions of 3 HUVEC-OGD and 3 HUVEC samples by the Illumina Novaseq 6,000 (Illumina, United States) after normalization. According to the screening criteria, 1,393 genes (including *HSPA6*, *ZFAND2A*, *CCL2*, *N4BP3,* and *F2RL3*) were significantly upregulated, and 1,466 genes (including *ID1*, *SMAD6*, *ZBTB16*, *SLC16A14*, and *TRIM8*) were downregulated in the HUVEC-OGD group (Figures 2B–E). To further explore these DEGs’ related pathways and biological functions, we performed GO and KEGG analyses (Figures 3A,B). Biological process (BP) analysis demonstrated most of these DEGs were related to the regulation of cell-cell adhesion (93 genes), peptidyl-tyrosine phosphorylation (92 genes), response to endoplasmic reticulum (ER) stress (74 genes), as well as response to topologically incorrect (63 genes) and unfolded (61genes) proteins. Cellular components (CC) categorisation showed that 112 DEGs were distributed in the extracellular matrix (ECM) and 87 in the cytoplasm. The molecular function (MF) analysis detected 103 DEGs that were relevant to receptor-ligand activities and 64 DEGs specific to the cytokine receptor binding function. Enrichments were also found in the KEGG analysis. Possible underlying pathomechanistic pathways of ischemia-hypoxia injury involved cytokine receptor signaling (74 genes), PI3K-Akt signaling (74 genes), hyperlipidemia and atherosclerosis (52 genes), and others.

To elaborate on the interaction between DEGs, we performed PPI network analyses on significant DEGs. 151 DEGs of top 1,000 exhibited interactions, which were divided into 42 clusters with 136 edges (Figure 3C). The top 20 hub genes were also closely related (Figure 3D).

### 3.2 Identification of OGD-associated DNA methylation positions

**FIGURE 4:**
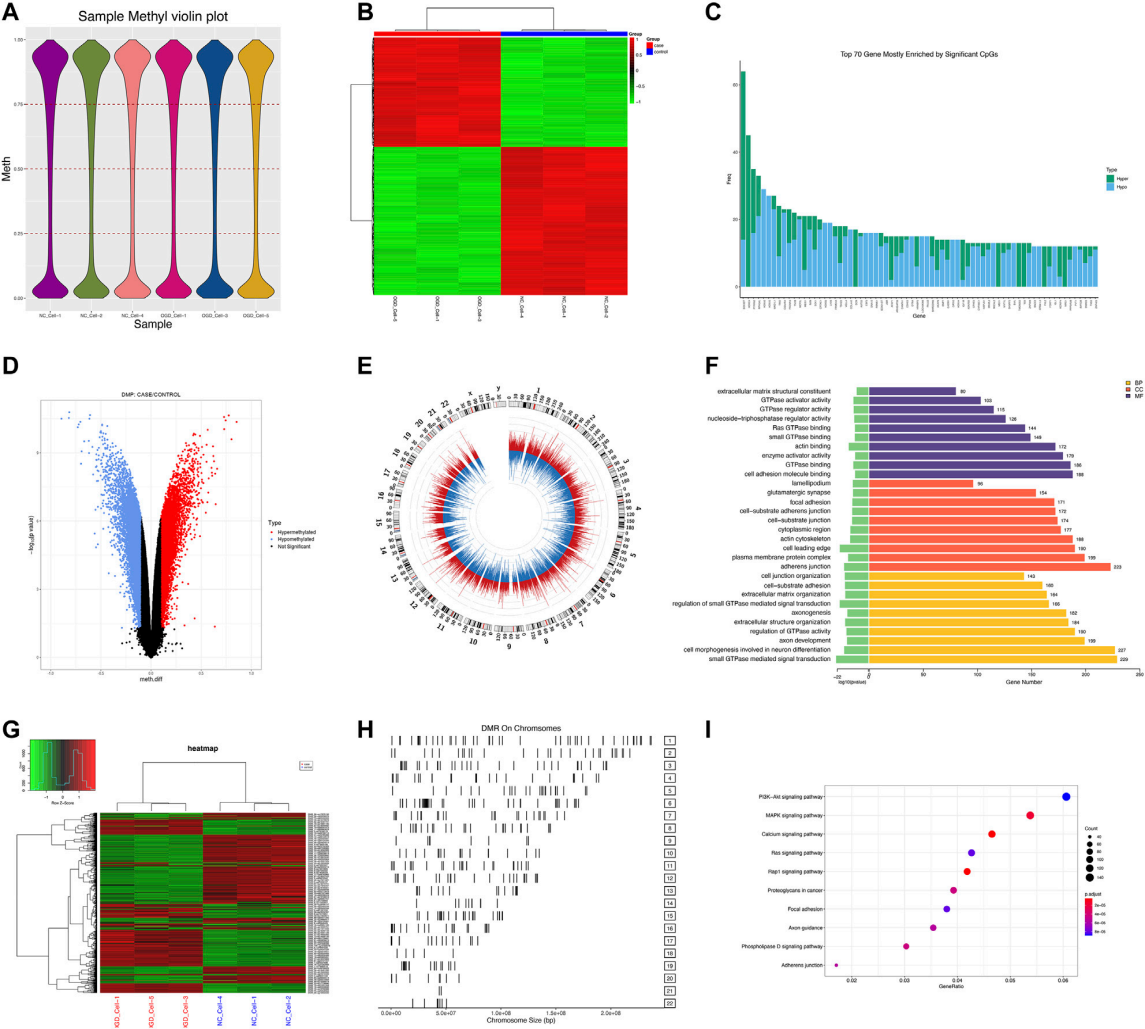
DMP and DMR analyses. **(A)** Violin plot for DNA methylation. The horizontal coordinate represents each sample and the vertical coordinate indicates the overall methylation level of that sample. **(B)** Heat map of top 1,000 DMPs. Each column represents a sample, and each row represents a gene where a DMP is located. **(C)** Bar plot of top 70 genes enriched for DMPs, where the horizontal coordinate indicates the single gene name, and the vertical coordinate shows the number of DMPs in that gene. **(D)** Volcano plot of DMPs. Red color for the hypermethylated OGD group relative to that of the control group, while blue color denotes hypomethylation. **(E)** Circle diagram of the chromosomal distribution of DMPs. The height of the bar inside the circle plot indicates |log2(FC)|, red color for the hypermethylated OGD group, and blue for the hypomethylated group. **(F)** Bar graph for GO enrichment of DMPs. **(G)** Heat map of top 100 DMRs. **(H)** Chromosomal distribution of DMRs. **(I)** KEGG scatter plot of DMPs.

We measured DNA methylation levels at 865,100 methylation sites in 3 HUVECs-OGD and 865,315 in 3 HUVEC controls using the Illumina Infinium MethylationEPIC BeadChip. After screening and QC check, 732,322 methylation positions were subjected to differential analysis. As shown in Figure 4A, the overall methylation levels of six samples were comparable. In total, 22,148 differentially methylated positions (DMPs; |Δβ|>0.1 and *p* = 0.0302), including 8,764 hypermethylated and 13,384 hypomethylated ones, were identified, which correctly separated most OGDs and NCs in the clustering analysis, as shown in the volcano plot of DMPs in Figure 4D **.** Locations of genome-wide distribution of differentially methylated CpG islands are described in Table 2. In contrast, 505 differentially methylated regions (DMRs) were identified among the 2,134 methylation regions examined. Of these, 276 were hyper- and 229 were hypo-methylated in the OGD group (Figure 4G). The differential analysis of DMPs and DMRs and their distribution levels in chromosomes are shown in Figures 4E,H. The heat map of top 1,000 DMPs and 100 DMRs are illustrated in Figures Figure4B,G. According to the annotation, 22,148 DMPs were physically located within 6,642 unique genes. Figure 4C indicates the top70 genes, including *SOX2OT*, *HOXC4*, *PTPRN2*, and *COL4A2*, are associated with numerous DMPs. Functional enrichment analyses showed that the 1844 genes were significantly enriched in some BP, most of which were related to OGD (Figure 4F). For example, 229 genes like *SQSTM1* and *NGFR* were enriched in GO:0007264/small GTPase-mediated signaling. Furthermore, the hub genes validated in the subsequent experiments also showed significant enrichment in pathways related to OGD, for instance, 167 DEGs were involved in GO:0050900/leukocyte migration, including COL1A2, TSP-1, VEGFA, CD44, and CCL2, and 194 DMGs were associated with GO:0198738/cell-cell Wnt signaling, including RUNX1, LRP5, and GNAQ. The enrichment was also found in KEGG pathways for OGD injury (e.g., hsa04151/PI3K−Akt signaling, hsa04010/MAPK signaling, hsa04510/focal adhesion, and hsa04020/calcium signaling) (Figure 4I). These results suggest that DNA methylation differences may play critical roles in the pathogenesis of ischemia and hypoxia in HUVECs.

**TABLE 2:** Distribution of genomic regions of significant differentially methylated CpG sites.

	Genomic region of CpG sites	All CpG sites,n (%)	Hypermethylated CpG sites,n (%)	Hypomethylated CpG sites,n (%)
Region-level gene based	TSS1500	2087	960 (46.00%)	1,127 (54.00%)
	TSS200	715	287 (40.14%)	428 (59.86%)
	5′-UTR	1783	597 (33.48%)	1,186 (66.52%)
	1st Exon	251	100 (39.84%)	151 (60.16%)
	Body	8,825	3,369 (38.18%)	5,456 (61.82%)
	ExonBnd	100	41 (41.00%)	59 (59.00%)
	IGR	7,928	3,217 (40.58%)	4,711 (59.42%)
	3′-UTR	459	193 (42.05%)	266 (57.95%)
Region-level island based	island	1,404	523 (37.25%)	881 (62.75%)
	N-shore	2070	813 (39.28%)	1,257 (60.72%)
	S-shore	1715	687 (40.06%)	1,028 (59.94%)
	N-shelf	752	228 (30.32%)	524 (69.68%)
	S-shelf	681	231 (33.92%)	450 (66.08%)
	opensea	15,526	6,282 (40.46%)	9,244 (59.54%)
total		22,148	8,764 (39.57%)	13,384 (60.43%)

^a^ CpG, 5′-C-phosphate-G-3'; UTR, untranslated region; TSS, transcription start site; N, north; S, south, and IGR, intergenic region.

### 3.3 Identified DMPs regulate mRNA expressions

**FIGURE 5:**
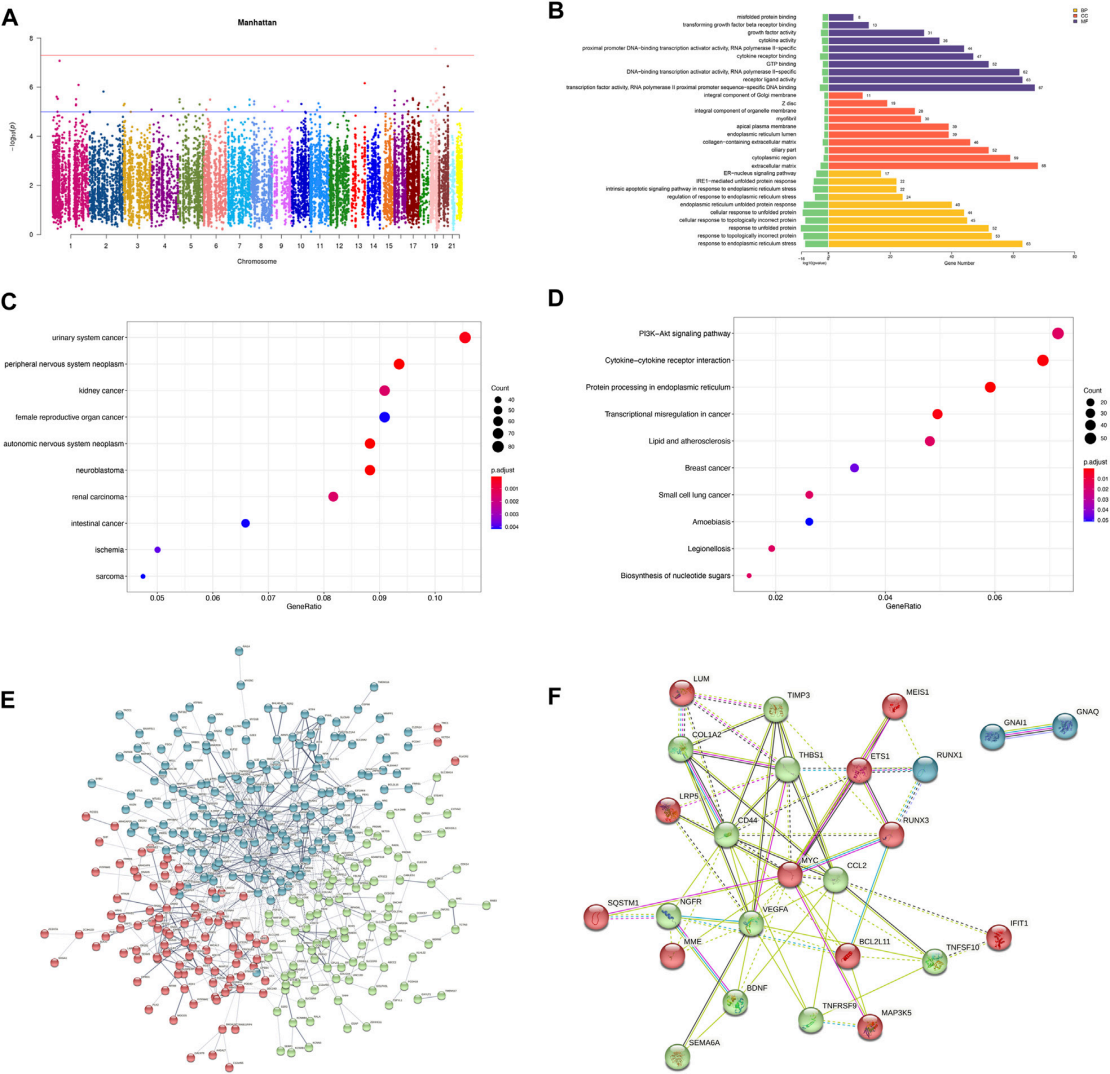
Analysis of DEGs associated with DMPs. **(A)** Manhattan plot of DEGs associated with DMPs. The horizontal coordinate indicates the chromosome and the vertical coordinate for the -log10 (*p*-value) of the DEGs associated with DMPs on the corresponding chromosome. **(B)** Bar graph of GO enrichment. **(C)** Scatter plot of DO. **(D)** Scatter plot of KEGG pathway enrichment. **(E)** PPI network constructed by the STRING using 441 DMGs and DEGs. The stronger associations are represented by thicker edges. The disconnected nodes are hided. Different colors represent different clusters. **(F)** PPI network constructed by the STRING using the top 25 hub genes (DMGs and DEGs).

Pearson correlation analysis showed that within 500 kb of upstream and downstream of differentially methylated CpG loci (Mendelson et al., 2018), there were 2,130 unique genes (covering 11,608 DMPs) with differentially expressed mRNA levels (*p* < 0.05; Pearson correlation coefficient>0.8). The distribution of DMPs associated with DEGs on the chromosome is shown in Figure 5A. Because correlation analysis alone does not establish causation, we conducted in-depth CIT analyses to investigate whether DNA methylation causes endothelial cell ischemic-hypoxic injury by regulating gene expression. In other words, we aimed to assess the potential causal chain of the causal factor (DNA methylation) - mediator (mRNA)—cell outcome (endothelial cell ischemic-hypoxic injury)-clinical outcome (AMI) (Zhu et al., 2019).which identified 1780 unique genes with 7,054 differentially methylated CpG loci. To investigate which pathophysiological processes were influenced by these 1780 DEGs, we conducted DO, GO term, and KEGG pathway enrichment analyses. DO enrichment analysis, including 759 genes, showed that kidney and nervous system cancer involved many of these genes, but still, there were 32 genes enriched in the DO (ID:326) of ischemia (*p* = 0.0038). GO term suggested there were 1,551 genes enriched in 984 functional categories (adjusted *p* < 0.05). The most considerable portion of the functional terms comprised BP (n = 845), while the rests were CC (n = 56) and MF (n = 83). When BPs were used for categorization, the majority of enriched groups included responses to ER stress, topologically incorrect protein stress, and unfolded protein-ER-nucleus signaling pathway. Categorization by CC indicated that proteins encoded by the target genes were mainly associated with the localization in the ECM and cytoplasm. The MF analysis demonstrated a significant gene enrichment in transcription factor (TF) activity, RNA polymerase II proximal promoter sequence−specific DNA binding, and receptor-ligand interaction. Figure 5B presents the top 10 terms of the three GO categories ranked by their statistical significance and scatter plot of DO is illustrated in Figure 5C. In the KEGG pathway analysis (Figure 5D), 727 genes showed significant enrichment in pathways, including PI3K−Akt signaling, cytokine receptor signaling, and ER signaling for protein processing. Further PPI analysis suggested 441 nodes and 770 edges, with an average node degree of 0.34, an average local clustering coefficient of 0.34, and an expected number of edges of 542 (PPI enrichment, *p* < 1.0e-16). These 441 genes were enriched in 75 BPs. For instance, 224 genes were enriched in GO: 0050896 (response to stimulus), 159 genes in GO: 0007154 (cell communication), 115 genes in GO: 0030154 (cell differentiation), 42 genes in GO:0016477 (cell migration), 41 genes in GO: 0007155 (cell adhesion), and 40 genes in GO: 0035295 (tube development). Furthermore, among the 6 enriched CCs, 38 genes were in GO: 0070161 (anchoring junction) and 27 genes in GO: 0005911 (cell-cell junction). These results indicated that DEGs in ischemic-hypoxic HUVECs were closely related to intercellular interactions and angiogenesis, where DNA methylation regulation played an important role. Figure 5E demonstrated the STRING analysis of 3 clusters of 441 DMGs.

### 3.4 Validation of key DMPs and corresponding DEGs

To verify the reliability of the sequencing results, we validated the top 25 hub genes in PPI analysis and their CpG islands in a new sample of the same OGD model. PPI analysis of these 25 genes was revealed in Figure 5F. Among them, *RUNX3*, *TNFRSF9*, and *MAP3K5* were not tested by qRT-PCR as we failed to construct any efficient and reproducible primer pairs, and no CpG islands were detected for genes *CCL2*, *IFIT1*, *LUM*, and *TNFSF10*.

**FIGURE 6:**
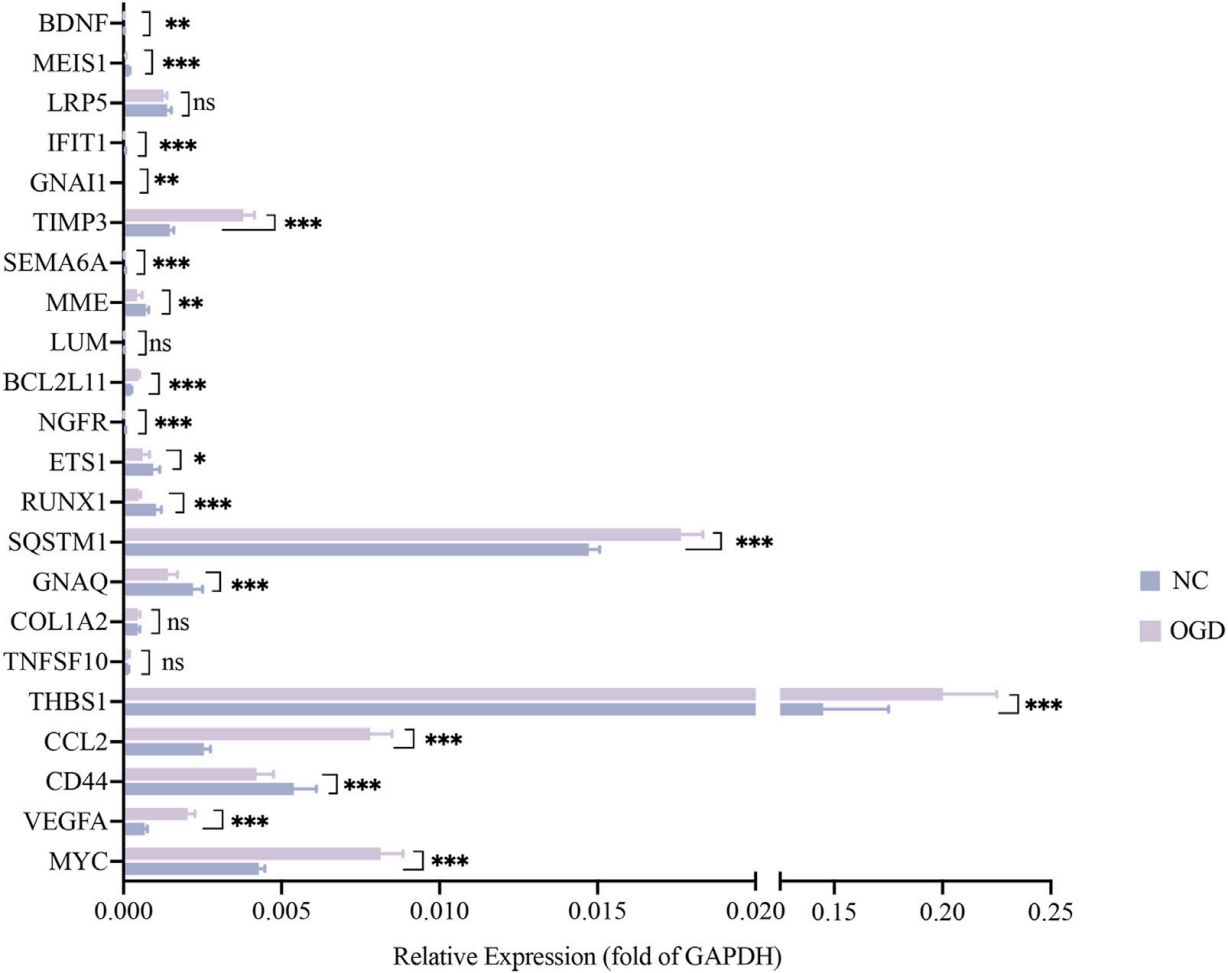
The mRNA expressions of 22 hub genes relative to GAPDH. The horizontal coordinates show each genes and the vertical coordinates for the expressions relative to GAPDH. Green color for the NC group and purple for the OGD group. *p < 0.05, **p < 0.01 and ***p < 0.001vs control group.

The results of qRT-PCR, the number of nodes in the PPI analysis, and the categories and examples involved in the functional clustering analysis of these 22 hub genes are listed in Table 3. The qRT-PCR analysis revealed that altered expressions of 12 DMGs were consistent with the sequenced samples, while 6 DMGs exhibited opposite expression patterns. Expressions of *COL1A2*, *LRP5*, *LUM*, and *TNFSF10* were not significantly changed (*p* > 0.05). As shown in Table 3 and Figure 6, expression levels of *VEGFA*, *CCL2*, *TSP-1*, *SQSTM1*, *BCL2L11*, and *TIMP3* were significantly elevated in the OGD group, while that of *MYC*, *CD44*, *BDNF*, *GNAQ*, *RUNX1*, *ETS1*, *NGFR*, *MME*, *SEMA6A*, *GNAI1*, *IFIT1*, and *MEIS1* decreased due to ischemia and hypoxic shocks.

**TABLE 3:** Expression and cluster analysis of 22 hub genes.

Gene	DEGs in sequencing			Validation by RT-qPCR				String node degrees	GO analysis		KEGG analysis	
	log2FoldChange	Type	p adjust value	Expression^a^		*p*-value	Type		Count	Example	Count	Example
				NC (%)	OGD (%)							
BDNF	1.251,956,305	Up	3.98264E-05	0.0023	0.0012	0.0015	Down	23	90	transport vesicle	9	MAPK signaling pathway
COL1A2	2.10,533,194	Up	5.0646E-12	0.0443	0.0457	0.4,461,868	Up	15	43	platelet activation	11	PI3K-Akt signaling pathway
TSP-1	1.272,757,804	Up	1.2377E-23	14.4803	20.0269	0.0006954	Up	19	323	response to decreased oxygen levels	12	p53 signaling pathway
MYC	1.025,573,653	Up	0.000118844	0.8137	0.4278	3.402E-06	Down	49	176	cellular response to hypoxia	33	Hippo signaling pathway
VEGFA	2.076,530,808	Up	5.89859E-14	0.0667	0.2030	1.77E-05	Up	39	359	regulation of transcription from RNA polymerase II promoter in response to hypoxia	23	HIF-1 signaling pathway
CD44	1.159,563,467	Up	0.001,341,815	0.5391	0.4210	3.694E-05	Down	34	113	leukocyte migration	6	ECM-receptor interaction
CCL2	5.069,944,423	Up	5.5462E-134	0.2544	0.7800	4.98E-07	Up	21	149	positive regulation of endothelial cell apoptotic process	17	NOD-like receptor signaling pathway
SQSTM1	1.548,462,125	Up	2.29785E-09	1.4727	1.7631	0.0004683	Up	15	85	autophagy of mitochondrion	9	Fluid shear stress and atherosclerosis
RUNX1	1.196,038,099	Up	3.15132E-12	0.1028	0.0477	1.66E-07	Down	14	125	positive regulation of angiogenesis	5	Tight junction
ETS1	1.606,366,428	Up	1.34887E-21	0.0904	0.0639	0.0165721	Down	13	87	response to hypoxia	4	Ras signaling pathway
BCL2L11	2.075,699,979	Up	3.44266E-28	0.0250	0.0469	3.648E-05	Up	11	163	tube formation	9	FoxO signaling pathway
MME	1.448,298,803	Up	9.58586E-06	0.0705	0.0430	0.0016804	Down	11	0		0	
TIMP3	1.64,760,662	Up	1.00799E-26	0.1460	0.3782	6.798E-07	Up	11	49	regulation of ERK1 and ERK2 cascade	2	MicroRNAs in cancer
GNAI1	−1.484,975,055	Down	1.19953E-14	0.0012	0.0004	0.0016735	Down	10	59	response to platelet aggregation inhibitor	40	Adrenergic signaling in cardiomyocytes
IFIT1	−2.936,485,257	Down	9.38353E-10	0.0057	0.0012	4.065E-06	Down	10	50	negative regulation of immune system process	1	Hepatitis C
LRP5	−1.469,642,161	Down	3.34967E-11	0.1383	0.1275	0.2,818,192	Down	10	113	tissue remodeling	8	Wnt signaling pathway
MEIS1	−1.76,793,772	Down	4.55049E-11	0.0180	0.0064	2.738E-05	Down	10	60	cardiac muscle tissue growth	2	Signaling pathways regulating pluripotency of stem cells
GNAQ	−1.068,236,204	Down	5.71031E-15	0.2201	0.1403	6.761E-05	Down	15	85	regulation of circadian rhythm	47	Rap1 signaling pathway
NGFR	−4.690,983,395	Down	1.67034E-55	0.0065	0.0008	4.48E-07	Down	13	87	regulation of blood vessel endothelial cell proliferation involved in sprouting angiogenesis	8	Apoptosis - multiple species
LUM	−2.996,325,065	Down	0.013,749,732	0.0023	0.0016	0.2,637,541	Down	11	40	regulation of transforming growth factor beta production	1	Proteoglycans in cancer
SEMA6A	−1.924,779,711	Down	1.381E-15	0.0056	0.0022	0.000511	Down	11	84	negative regulation of cell adhesion	1	Axon guidance
TNFSF10	−1.268,836,896	Down	1.24894E-07	0.0143	0.0148	0.667154	Up	16	45	apoptotic mitochondrial changes	10	Cytokine-cytokine receptor interaction

^a^ Expression relative to GAPDH.

**FIGURE7:**
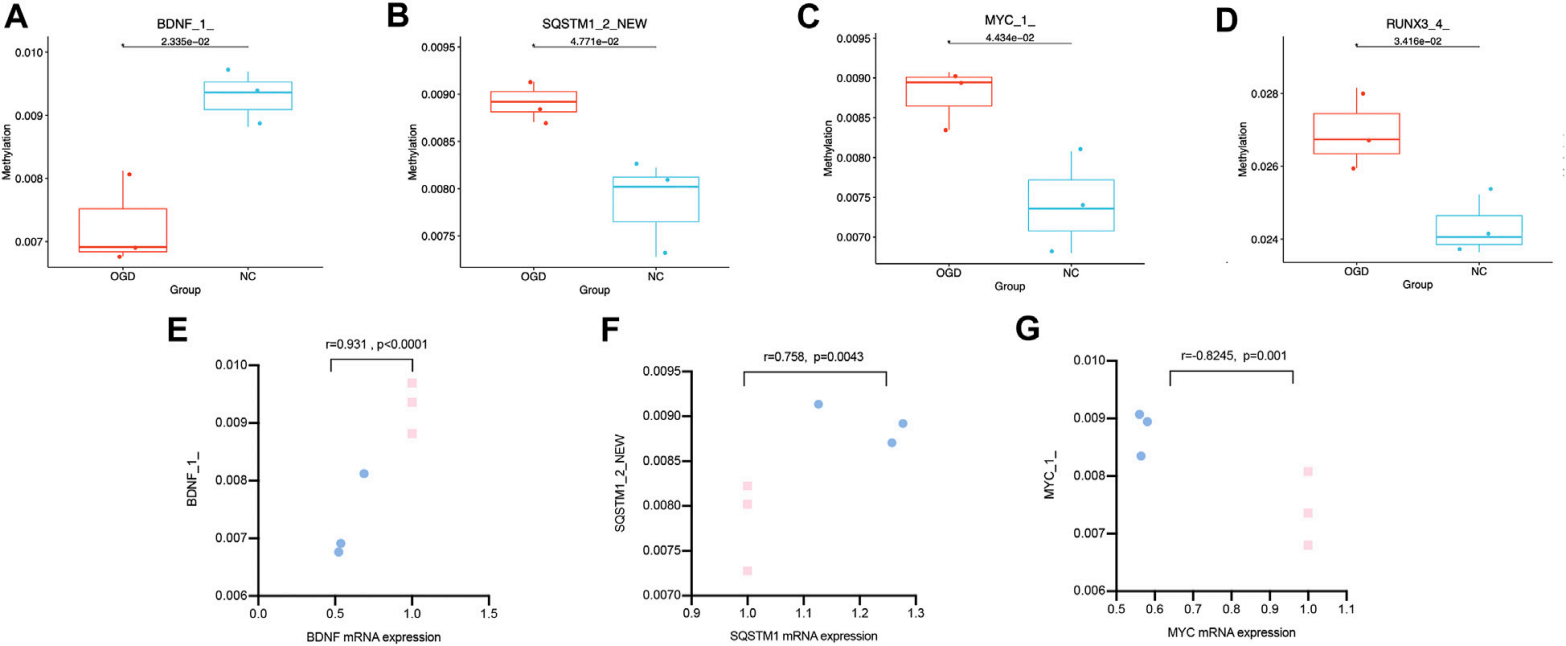
Differentially expressed CpG fragments and correlation analysis. **(A–D)** The horizontal coordinates represent each groups and the vertical coordinates represent the methylation levels. Each point represents each sample, and the box plot illustrates the median and quartiles. **(E–G)** The horizontal coordinates represent the relative mRNA expression levels, and the vertical coordinates represent the methylation levels of methylated fragments. The pink squares represent the NC group, while the blue dots represent the OGD group.

We quantified the methylation levels of CpG islands and DNA fragments from 21 DMGs by MethylTarget sequencing. Notably, 42 of 1,179 CpG sites were differentially expressed, and 15 of 486 methylated haplotypes were significantly different in abundance (see Table 2; Table 3 in the supplement). Four of the 66 fragments were differentially expressed: BDNF_1_demethylated in the OGD group (*p* = 0.02334636), MYC_1_ (*p* = 0.0443373), RUNX3_4_ (*p* = 0.03415566) and SQSTM1_2_NEW (*p* = 0.04771069) had higher methylation levels than before (Figures 7A–D), where fragments BDNF_1_ (r = 0.931, *p* < 0.0001) and SQSTM1_2_NEW (r = 0.758, *p* = 0.0043) were positively correlated with the mRNA expressions of corresponding genes, and MYC_1_ (r = −0.8245, *p* = 0.001) was negatively correlated with the mRNA expression (Figures 7E–G).

### 3.5 BDNF and TNFSF10 overexpresses in AMI patients

**FIGURE 8:**
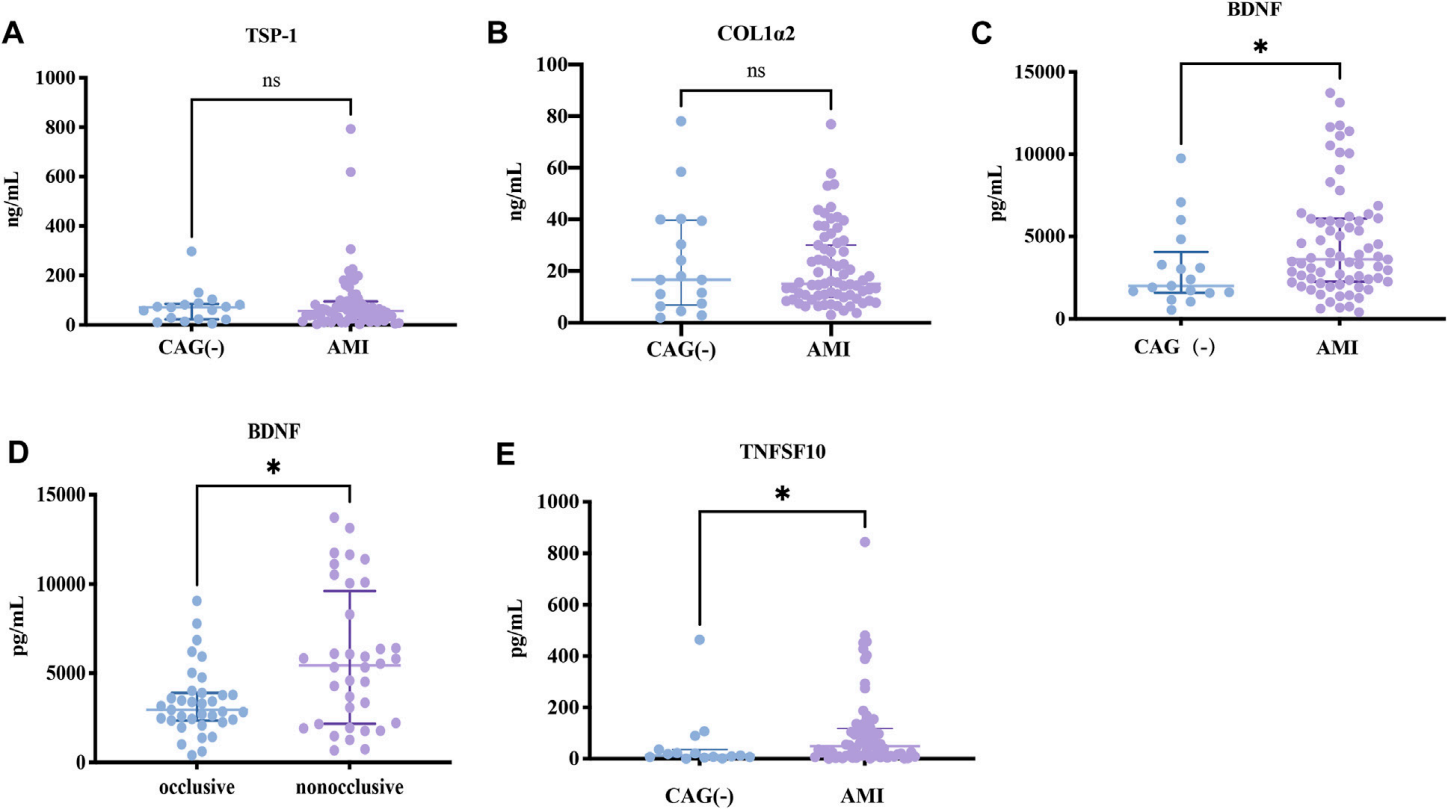
ELISA results about key candidates. **(A,B)** There were no differences in expressions of TSP-1 and COL1α2 between AMI and NC patients (*p* > 0.05). **(C,D)** BDNF and TNFSF10 were overexpressed in AMI patients (*p* = 0.0284 and *p* = 0.0142, respectively). **(E)** BDNF expression was lower in the occlusion group compared to the non-occlusion group (*p* = 0.0118).

Since the expressions of target genes were significantly altered in endothelial cells during ischemia and hypoxia, we hypothesized that there might have corresponding changes in coronary artery endothelial cells in the AMI patients and if disease-related factors released into the blood might be exploited as biomarkers. Based on previous findings and the differential expression analyses, we selected four indicators to be tested in downstream experiments. The basic clinical characteristics of the patients are described in Table 4. ELISA test results revealed that BDNF and TNFSF10 levels were indeed elevated in the peripheral blood of AMI patients (Table 5; Figure 8 C&E), and the BDNF expression was slightly lower in the group with complete occlusion under coronary angiography, compared with the group with non-complete occlusion [2,962 (2,362, 3,909) vs. 5,347 (2048, 9,181); *p* = 0.0204; Figure 8 D ]. Correlation analysis showed that TNFSF10 was positively correlated with the expression of homocysteine, COL1α2 and BDNF, while negatively correlated with EF, suggesting that TNFSF10 might be associated with heart failure. BDNF, in turn, was positively correlated with TSP-1 level and platelet and leukocyte count. In contrast, although COL1α2 and TSP-1 expressions could not be detected among differentially expressed genes in the AMI group, correlation analysis revealed that the expression of COL1α2 was positively correlated with the length of time from the disease onset to a blood draw and negatively correlated when patients took a double antiplatelet prior to phlebotomy. TSP-1, on the other hand, was positively correlated with NT-proBNP expression (Table 6).

**TABLE 4:** The clinicopathological features of candidates.

	Group		*p*-value
	CAG(−) (n = 17)	AMI (n = 71)	
Age, years (medium (25% Percentile, 75% Percentile))	56 (52, 68.5)	59 (54, 69)	>0.05
Male (%)	10 (62.5%)	65 (90.3%)	0.005
BMI (mean ± SD)	23.87 ± 2.509	25.66 ± 5.461	>0.05
Family history (%)	1 (5.9%)	17 (18.3%)	>0.05
History of hypertension (%)	5 (29.4%)	38 (53.5%)	>0.05
History of diabetes (%)	5 (29.4%)	10 (14.1%)	>0.05
History of hyperlipidemia (%)	0 (0.0%)	1 (1.4%)	>0.05
History of drinking (%)	1 (5.9%)	12 (16.9%)	>0.05
History of Smoking (%)	3 (17.6%)	36 (50.7%)	0.014
History of COPD(%)	1 (5.9%)	1 (1.4%)	>0.05
History of cardiovascular and cerebrovascular disease (%)	0 (0.0%)	6 (8.5%)	>0.05
Systolic pressure, mmHg	131.80 ± 19.95	133.30 ± 25.63	>0.05
Diastolic pressure, mmHg	77.88 ± 8.97	74.58 ± 13.66	>0.05
HR, bpm	79.06 ± 19.32	72.42 ± 13.97	>0.05
TNI, ng/mL	0.01 (0.01,0.01)	2.4 (0.94,16)	<0.0001
MYO, ng/mL	55.76 ± 28.01	335.7 ± 217.4	0.0005
CKMB, ng/mL	2 (2, 2.4)	45.46 (15.28, 177)	<0.0001
d-Dimer, ug/mL	0.25 (0.17, 0.6555)	0.339 (0.17, 2.07)	>0.05
NT-proBNP, pg/mL	64 (37, 598)	617.5 (234.3, 1,363)	0.0025
Leukocytes, 10–9/L	6.346 ± 1.829	10.41 ± 3.4	<0.0001
Hb, g/L	147 (141.5, 162)	161 (147, 168)	>0.05
NE%, %	70.7 (61.75, 73.8)	81.3 (74.5, 84.9)	<0.0001
Platelet, 10–9/L	175 (111, 204)	183 (161, 222)	>0.05
Creatinine, ummol/L	78.21 ± 25.2	71.75 ± 15.78	>0.05
Uric acid, ummol/L	343.9 ± 101.1	347.6 ± 86.74	>0.05
Total cholesterol, mmol/L	3.975 ± 0.8251	4.599 ± 1.011	0.0205
Triglycerides, mmol/L	1.55 (1.065, 3.185)	1.66 (1.12, 2.34)	>0.05
HDL-C, mmol/L	1.052 ± 0.364	1.018 ± 0.2294	>0.05
LDL-C, mmol/L	2.462 ± 0.5984	3.089 ± 0.7789	0.0026
APOAI, g/L	1.24 ± 0.3082	1.18 ± 0.2245	>0.05
APOB, g/L	0.8482 ± 0.3331	0.9909 ± 0.2654	>0.05
APOB/AI	0.68 (0.52, 0.79)	0.82 (0.655, 1.015)	0.0105
LP(a), mg/dL	7.53 (4.27, 31.27)	12.62 (5.615, 26.64)	>0.05
Hcy, umol/L	14.6 (10.6, 24.75)	16.5 (12.55, 22.5)	>0.05
Glycohemoglobin, %	5.7 (5.3, 7.1)	5.7 (5.225, 6.15)	>0.05
PT, s	11.4 (10.95, 12.75)	11.9 (11.5, 12.5)	>0.05
APTT, s	31.8 (29.5, 34.7)	33.4 (30.1, 44.3)	>0.05
FDP, ug/mL	0.9 (0.77, 1.53)	1.15 (0.69, 3.45)	>0.05
EF, %	62 (58.5, 64.5)	55 (50, 58)	0.0001
**Infarct related artery**			
LM		2 (2.8%)	
LAD		30 (42.3%)	
LCX		8 (11.3%)	
RCA		31 (43.7%)	
**number of stenosed coronary vessel**			
1		31 (43.7%)	
2		23 (32.4%)	
3		17 (23.9%)	
**Onset-blood drawing time**			
up to 6 h		28 (39.44%)	
7-12 h		24 (33.80%)	
over 12 h		19 (26.76%)	
coronary occlusion in angiography		38 (53.52%)	
Thrombolytic therapy		17 (23.94)	

**TABLE 5:** Expression of BDNF, TNFSF10,TSP-1 and COL1α2 in AMI and control patients.

Variable	Group		*p*-value
	Control (n = 17)	AMI (n = 71)	
BDNP (pg/mL)	2011 (1,597, 4,053)	3,609 (2,256, 6,082)	0.0284
TNFSF10 (pg/mL)	13.18 (6.875, 36.51)	48.46 (11.65, 117.7)	0.0309
TSP-1 (ng/mL)	71.17 (22.93, 85.38)	56.79 (25.79, 95.66)	>0.05
COL1α2 (ng/mL)	16.62 (6.912, 39.71)	15.09 (9.836, 30.04)	>0.05

**TABLE 6:** Correlation analysis of key indicators.

Variable	Variable	Pearson’s correlation coefficient	*p*-value
TNFSF10	Hcy	0.294	0.006
TNFSF10	COL1α2	0.281	0.008
TNFSF10	BDNF	0.563	<0.001
TNFSF10	EF	−0.274	0.01
TSP-1	BDNF	0.293	0.006
COL1α2	Onset-blood drawing time	0.317	0.007
COL1α2	Dual antiplatelet before blood drawing	−0.256	0.03
BDNF	platelet	0.246	0.021
BDNF	WBC	0.223	0.037
TSP-1	NT-proBNP	0.264	0.018

## 4 Discussion

Acute myocardial infarction (AMI) is a complication of atherosclerosis that takes place in the coronary arteries (Xiao et al., 2020). It causes severe damage to the coronary microcirculation, resulting in vascular disintegration and capillary thinning in the infarct zone. Cardiac endothelial cells are estimated to be almost triple the number of cardiomyocytes (Pinto et al., 2016; Xiao et al., 2020). Endothelial dysfunction is involved from the initial stage of atherosclerosis to the late stage of cardiovascular complications (Gimbrone and Garcia-Cardena, 2016). Additionally, it serves as a marker of cardiovascular risk and a contributor to the progression of cardiovascular events. What is more, cardiac endothelial cells play a vital role in remodeling injured cardiac myocytes after cardiac tissue injury (Segers et al., 2018). Tissue repair following the myocardial infarction involves a drastic angiogenic response that begins in the infarction border zone and extends to the necrotic infarct core. Cell lineage tracing studies have revealed that new capillary structures are generated by angiogenesis only from pre-existing endothelial cells in the infarction border zone (Zhou et al., 2011; Dube et al., 2017; Tang et al., 2018). As a key factor in the function of blood vessels, the viability of endothelial cells is decisive in the reconstruction of blood flow for rescuing cardiomyocytes, reducing infarct size, and improving cardiac function (Guo et al., 2021). Furthermore, animal studies also support the notion that post-AMI endogenous angiogenic responses can be boosted to reduce scarring and adverse left ventricular remodeling (Wu et al., 2021).

The combination of LAD ligation and the OGD model is commonly used *in vivo* and *in vitro* studies of AMI (Xiao et al., 2020; Wang et al., 2021; Guo et al., 2021; Zhang et al., 2021; Wu et al., 2022). As early as 2000, researchers used the OGD model to simulate oxidative stress in myocardial cells under ischemic and hypoxic conditions (Persky et al., 2000). OGD/R-induced endothelial cytotoxicity alters the cellular pH balance, increases oxidative stress, and reduces endothelial nitric oxide production (Yang et al., 2016). These changes are inseparable from the regulation of DNA methylation. Therefore, to explore the gene expression profiles of endothelial cells under ischemic-hypoxic stress and reveal the regulatory roles of DNA methylation on the mRNA expression, facilitating further exploration of early diagnosis of AMI and therapeutic targets in post-AMI angiogenesis, we utilized the HUVEC OGD model to simulate *in vitro* ischemic-hypoxic conditions in endothelial cells.

Firstly, We performed sequencing and validation to identify a large number of DEG and differentially methylated loci. Among them, fragment BDNF_1_ was demethylated, and BDNF gene expression was decreased in response to ischemia and hypoxic shocks. SQSTM1_2_NEW methylation level and SQSTM1 gene expression were both elevated. Hypermethylation of MYC_1_ did cause a decrease in MYC gene expression. In addition, we found that TNFSF10 and BDNF proteins were differentially elevated in the peripheral serum of AMI patients. The inconsistent trends in the expression of target molecules measured in cells and patient serum were also well understood. Levels of target molecules in the cellular assays only represented the local distribution in HUVECs. On the contrary, these factors represented their total amounts corresponding to overall tissues and organs In the serum.

Brain-derived neurotrophic factor (BDNF), a member of the neurotrophic factor family, is involved in stress and inflammation. As an essential component of ischemic tissue angiogenesis, BDNF can stimulate the migration and proliferation of ischemic local endothelial cells mediating cell survival (Pius-Sadowska and Machalinski, 2017). Recently, it has been discovered that vascular smooth muscle cells, endothelial cells, and atherosclerotic arteries express BDNF (Ejiri et al., 2005). A study (Donovan et al., 2000) using animal models showed that endothelial cell survival in intramyocardial arteries and capillaries during the early postnatal period could be compromised by low levels of BDNF expression. Insufficient BDNF can cause intraventricular wall bleeding, decreased heart contractility, and early postnatal mortality because of reduced endothelial cell-cell interactions and increased apoptosis. In clinical research, Luigi Manni et al. (Manni et al., 2005) found that serum BDNF levels decreased in acute coronary syndromes, but the number of cases in this study was very minimal (n = 31). On the contrary, Haibo Wu et al. (Wu et al., 2019) reported that serum BDNF expressions were higher in AMI patients combined with AHF than in AMI patients without AHF. Shinpei Kadowaki et al. (Kadowaki et al., 2016) demonstrated that serum BDNF levels in 134 chronic heart failure patients were significantly lower than that of 23 control subjects. In another study (Ejiri et al., 2005), the difference in BDNF levels between the coronary sinus and aorta was significantly greater in the unstable angina group compared with the stable angina and non-coronary artery disease groups. A review that included 35 studies showed that BDNF levels were lower in patients with chronic heart failure and stroke, but higher in patients with unstable angina and myocardial infarction. Our finding match those observed in earlier studies that BDNF expression was significantly higher in the AMI group, but we performed subgroup analysis and found no significant difference between the ST-segment elevation myocardial infarction (STEMI) and NSTEMI groups, while was significantly lower in the completely occluded group than in the non-occluded group at coronary angiography. Moreover, serum BDNF levels correlated with the number of platelets in peripheral blood since circulating BDNF was captured and sequestered by circulating platelets, consistent with previous studies (Yang et al., 2006; Farmer et al., 2021). Considering the relationship between BDNF expression and inflammation, it is reasonable to suggest that there could be a link between BDNF level and white blood cell count. Hence, we suspected the elevated BDNF expression in the AMI group might be related to rapid endothelial cell proliferation and revascularization after ischemia.

Tumor necrosis factor superfamily member 10 (TNFSF10), also known as tumor necrosis factor-related apoptosis inducing ligand (TRAIL), is a soluble marker of apoptosis. TNFSF10 is normally a membrane-bound ligand expressed by immunocytes. Soluble TNFSF10 can also act as a weaker inducer of apoptosis compared to membrane-bound TNFSF10 (Li et al., 2003). The significance of TNFSF10 in endothelial cells has been studied. Jie Hui Li et al. (Li et al., 2003) showed that incubation of endothelial cells with TNFSF10 induces inflammation and apoptosis. In surviving cells, TNFSF10 promotes the adhesion of leukocytes. Injection of TNFSF10 into human skin xenografts promotes focal EC injury accompanied by limited neutrophil infiltration. Also, TNFSF10 protects endothelial cells from apoptosis and proliferation through the activation of Akt and extracellular signal-regulated kinase (ERK) pathways, partly due to nitric oxide generation (Wu et al., 2021; Wu et al., 2022). Several studies (Secchiero et al., 2009; Osmancik et al., 2013; Teringova et al., 2018) revealed that TNFSF10 level was lower after PCI in AMI patients compared to a pre-procedure or healthy population and gradually increased after that. A low TNFSF10 level is an indicator of heart failure and poor prognosis (Teringova et al., 2018). Nakajima et al. (Nakajima et al., 2003) indicated that the expression of TNFSF10 on peripheral lymphocytes in AMI patients increases compared with healthy controls. This study also reported that TNFSF10 protein expression was higher in human atherosclerotic plaques, especially the most vulnerable ones, and could be induced by the ox-LDL expression. Soluble active TNFSF10 negatively regulates calcium influx through store-operated calcium release-activated calcium channels, which is crucial to activating lymphocytes (Lunemann et al., 2002). Our sequencing results in endothelial cells showed that TNFSF10 was significantly downregulated during ischemia and hypoxia and slightly elevated upon re-modeling verification. Whereas analysis of blood samples of AMI patients before PCI demonstrated that the level of soluble TNFSF10 was elevated, and its expression was positively correlated with that of BNDF and COL1α2. This contradictory conclusion may be related to the involvement of different types of receptors. There are five types of TNFSF10 receptors: TRAIL-RI (DR4), TRAIL-R2 (DR5), TRAIL-R3 (DcRl), RAIL-R4 (DcR2), and osteoprotegerin (OPG) (Buchsbaum et al., 2006)^.^ The first two are death receptors, and the last three are decoy receptors, playing a role in promoting and inhibiting apoptosis, respectively. In this study, TNFSF10 was not found to correlate with LDL, ApoAI, APOB, and LPa, but we did not detect the ox-LDL level. This study further suggests that TNFSF10 correlates with EF and Hcy, and is needed to explore the mechanisms involved.

Collagen type 1 alpha 2 (COL1α2), a major component of fibrotic tissue and associated with excessive collagen production, is less studied in the heart, rather than that in the kidney. In diabetic nephropathy studies, excessive aggregation of COL1α2 has been associated with renal fibrosis (Das et al., 2022). In clinical investigations, increased levels of COL1α2 have also been associated with inflammatory fibrosis (Wang et al., 2021). Studies have shown that COL1α2 expression is directly regulated by HIF-1α binding to a functional hypoxia-responsive element in its promoter at −335bp relative to the transcription start site (TSS). Phosphorylated Smad3 also associates with the −335 hypoxia-responsive element of the COL1α2 promoter region independent of a Smad DNA binding sequence (Baumann et al., 2016). Hypoxia simultaneously stimulates ECM synthesis and suppresses its turnover due to increased production of COL1α2, decreased collagenase expression, and increased tissue inhibitor of metalloproteinase (TIMP)-1 (Norman et al., 2000)^.^ In terms of COL1α2 in heart research, a bioinformatics analysis revealed that COL1α2 underlies the comorbidity mechanisms of HF and depression (Huang et al., 2022). Single-cell sequencing results using an obese mouse model suggested that COL1α2 and COL1α1 might be important markers of obesity-induced cardiac fibrosis (Pan et al., 2022)^.^ Another investigation confirmed the association between COL1α2 and cardiac fibrosis (Xu et al., 2021). In our study, COL1α2 was overexpressed in the OGD group. Although any significant increase in expression was not observed in the peripheral blood of AMI patients, the analysis showed a positive correlation between the expressions of COL1α2 and TNFSF10, and the time from onset to blood sampling, and a negative correlation with the administration of dual antiplatelet agents, suggesting a gradual initiation of fibrosis with prolonged ischemia. Based on these findings, we measured the COL1α2 level in endothelial cells, indicating that endothelial cells could be involved in the post-ischemic myocardial fibrosis processes and that the administration of antiplatelet agents would attenuate the degree of fibrosis.

Thrombospondin-1 (TSP-1 or THBS-1) is a significant component of platelet granules and a thrombin-sensitive ECM glycoprotein (McLaughlin et al., 2005), that produces adaptive ER stress through interaction with activating transcription factor 6α (ATF6α). Increased expression of TSP-1 has been reported to be associated with thrombosis (Vallejo et al., 2000), which is significantly elevated in large vessels with atherosclerotic lesions (Smadja et al., 2011), peripheral arterial diseases (Huang et al., 2015), as well as AMI (Abdelmonem et al., 2017). The pathophysiological mechanisms may include upregulation of platelet aggregation, adhesion of endothelial cells and leukocytes (Narizhneva et al., 2004), chemotaxis and proliferation of VSMCs (McLaughlin et al., 2005; Krishna and Golledge, 2013), reduction of the physiological protective effects of nitric oxide (NO) (Rogers et al., 2014), impact on angiogenesis, and expression of cell adhesion factors that play crucial roles in inflammation and atherosclerosis (Krieglstein and Granger, 2001). Studies have shown that thrombin can not only induce platelet activation and regulate TSP-1 by releasing granules but also modulate the expression of TSP-1 in endothelial cells (McLaughlin et al., 2005). Yang Xiang et al. (Xiang et al., 2022) found that elevated levels of TSP-1 and BNP in patients with chronic heart failure and TSP-1 expression were significantly correlated with alterations in cardiac functions. Our study revealed that the TSP-1 mRNA level was significantly elevated in the OGD treatment. However, there was no overexpression of TSP-1 in the serum of AMI patients. Further expansion of sample size and refinement of blood sampling time may lead to more objective experimental results. Nevertheless, the expression of TSP-1 was positively correlated with the serum levels of BDNF and NT-proBNP. In summary, TSP-1 might not only be related to plaque formation but also play an essential role in heart failure and may also have a mechanistic connection with TSP-1 expression in promoting the release of tumor necrosis factor-alpha (TNF-α) from macrophages (Lopez-Dee et al., 2011; Li et al., 2013).

Furthermore, among the validated hub gene products, MYC binds to the VEGFA promoter region to activate VEGFA expression and subsequent sprouting of angiogenesis (Shi et al., 2014). Studies in tumor tissues have indicated that CD44 plays a role as a cell surface receptor in processes like cell-cell interaction, adhesion, and migration, thereby facilitating the sensing and immune response to pathological lesions in the tumor microenvironment (Yoshida et al., 2012). CCL2 may be involved in the recruitment of monocytes into the arterial wall during the progression of atherosclerosis (Li et al., 1993). GNAQ (a G-protein subunit alpha q) is required for platelet activation, and its mutation in endothelial cells leads to capillary malformations (Couto et al., 2016). ETS1, as a transcription factor, can regulate angiogenesis by modulating the expression of genes controlling endothelial cell migration and invasion (Yordy et al., 2005). Semaphorin 6A (SEMA6A), as a cell surface receptor for PLXNA2, plays an important role in cell-cell signaling as well as promotes the reorganization of the actin cytoskeleton (Perez-Branguli et al., 2016)^.^ TIMP metallopeptidase inhibitor 3 (TIMP3) is an antagonist of the matrix metalloproteinases, a group of peptidases involved in the degradation of the ECM. TIMP3 was reduced in various cardiovascular diseases, and study had shown that TIMP3 replenishment ameliorates the disease, suggesting a therapeutic potential for TIMP3 in cardiovascular diseases (Fan and Kassiri, 2020)^.^ Nerve growth factor receptor (NGFR), also known as TNF receptor superfamily member 16 (TNFRSF16), binds to BDNF (Tapia-Arancibia et al., 2004). Lumican (LUM), membrane metalloendopeptidase (MME), G protein subunit alpha i1 (GNAI1), interferon-induced protein with tetratricopeptide repeats (1IFIT1), LDL receptor-related protein 5 (LRP5), and Meis homeobox 1 (MEIS1) was barely studied in the ischemic-hypoxic endothelial cells or AMI, even though qRT-PCR confirmed that the expressions of *NGFR*, *LUM*, *SEMA6A*, *GNAI1*, and *IFIT1* were relatively lower. Specific roles played by these factors and the regulatory pathways in the OGD-treated HUVECs can further be investigated *in vivo* models.

Our study focused on the importance of endothelial cells in AMI, aimed to establish the potential causal chain of the causal factor (DNA methylation) - mediator (mRNA)—cell outcome (endothelial cell ischemic-hypoxic injury)-clinical outcome (AMI), and the findings laid a solid foundation for screening essential diagnostic and prognostic biomarkers of coronary endothelial cell injury of AMI. Secondly, we combined the sequencing results from *in vitro* cell experiments with clinical samples to demonstrate the feasibility of cellular assay screening and *in vivo* validation. Furthermore, our study provided the first evidence that during ischemia and hypoxia, the expression of BNDF was regulated by DNA methylation in endothelial cells and elevated in peripheral blood. Our study also had some shortcomings that were worth improving. Firstly, Hi-C experiments should be employed to elucidate the genes associated with DNA methylation based on the physical interaction. Moreover, the lack of validation regarding the specific pivotal role of BDNF, is a question we need to address. Last but not least, the clinical significance of the screened target proteins could be further explored by drawing blood from the coronary circulation before and after the primary PCI in the future.

## Data Availability

The data generated in this study, including RNA-seq and DNA methylation chip are deposited at https://www.ncbi.nlm.nih.gov/bioproject/PRJNA1035287 and https://www.ncbi.nlm.nih.gov/bioproject/PRJNA934412.

## Abbreviations

GO, gene ontology; KEGG, Kyoto Encyclopedia of Genes and Genomes; DO, disease ontology; MSP, methylation-specific polymerase chain reaction; TSS, transcription start site; UTR, untranslated region; AMI, acute myocardial infarction; HUVECs, human umbilical vein endothelial cells; OGD, Oxygen-Glucose Deprivation; DEGs, differentially expressed gens; DMPs, differentially methylated positions; DMGs, differentially methylated genes; DMRs, differentially methylated regions; CpG, 5′-C-phosphate-G-3′; CIT, Causal Inference Test; PPI, protein-protein interaction network; BDNF, Brain-derived neurotrophic factor; TNFSF10, Tumor necrosis factor superfamily member 10.
