# Supplementary material for: Methylation and transcriptomic expression profiles of HUVEC in the oxygen and glucose deprivation model and its clinical implications in AMI patients
Source: Front Genet. 2023 Dec 7;14:1293393. doi: 10.3389/fgene.2023.1293393 (PMC10740152; doi:10.3389/fgene.2023.1293393)
Supplement: Supplementary file 1 [file Table1.docx]

Supplementary Material

# Supplementary Table 1

# Defferently methylated CpG loci on 23 Hub DEGs in methylation sequencing

| **probe** | **CHR** | **UCSC_RefGene_Group** | **UCSC_RefGene_Name_Annovar** | **OGD_mean** | **NC_**  **mean** | **Fold**  **Change** | **meth.diff** | **P.Value** | **Type** |
| --- | --- | --- | --- | --- | --- | --- | --- | --- | --- |
| cg18775580 | 10 | IGR | IFIT3(dist=7620),IFIT1B(dist=29468) | 0.594977571 | 0.72710825 | 0.818279218 | -0.13213068 | 4.22887E-04 | Hypomethylated |
| cg23037265 | 10 | TSS1500 | IFIT3(dist=36028),IFIT1B(dist=1060) | 0.3922261 | 0.883518832 | 0.443936321 | -0.491292732 | 2.88972E-09 | Hypomethylated |
| cg05552874 | 10 | Body | IFIT1 | 0.861842547 | 0.695913302 | 1.238433788 | 0.165929245 | 6.15575E-05 | Hypermethylated |
| cg18354203 | 11 | Body | BDNF-AS | 0.880714998 | 0.777691176 | 1.13247395 | 0.103023822 | 1.19702E-04 | Hypermethylated |
| cg25928860 | 11 | Body | BDNF-AS | 0.306148922 | 0.18138968 | 1.687796798 | 0.124759241 | 0.002425969 | Hypermethylated |
| cg11865360 | 11 | TSS1500 | BDNF | 0.33319541 | 0.46247382 | 0.720463289 | -0.12927841 | 8.93538E-05 | Hypomethylated |
| cg04106006 | 11 | Body | BDNF | 0.124705727 | 0.270656891 | 0.460752087 | -0.145951163 | 4.09402E-04 | Hypomethylated |
| cg06639296 | 11 | IGR | BDNF(dist=55291),KIF18A(dist=243298) | 0.785285375 | 0.624692818 | 1.257074441 | 0.160592557 | 4.76102E-06 | Hypermethylated |
| cg04242343 | 11 | IGR | BDNF(dist=85791),KIF18A(dist=212798) | 0.50388435 | 0.343674089 | 1.466169159 | 0.160210261 | 3.85885E-04 | Hypermethylated |
| cg14082886 | 11 | Body | CD44 | 0.579396391 | 0.753055801 | 0.769393702 | -0.17365941 | 4.33044E-04 | Hypomethylated |
| cg04171808 | 11 | Body | CD44 | 0.604308492 | 0.705336304 | 0.856766466 | -0.101027812 | 4.40225E-05 | Hypomethylated |
| cg15427520 | 11 | 3'UTR | CD44 | 0.339184663 | 0.185283807 | 1.830622265 | 0.153900855 | 1.85700E-06 | Hypermethylated |
| cg15829423 | 11 | Body | LRP5 | 0.493950039 | 0.827994358 | 0.596562083 | -0.334044319 | 1.77704E-07 | Hypomethylated |
| cg06341100 | 11 | Body | LRP5 | 0.316322685 | 0.500359293 | 0.632191086 | -0.184036608 | 2.10248E-07 | Hypomethylated |
| cg12289926 | 11 | Body | LRP5 | 0.838844088 | 0.680966193 | 1.231843954 | 0.157877895 | 3.50885E-06 | Hypermethylated |
| cg18935453 | 11 | Body | LRP5 | 0.464286 | 0.578127354 | 0.803086026 | -0.113841355 | 2.81195E-04 | Hypomethylated |
| cg06210783 | 11 | Body | LRP5 | 0.614346659 | 0.756480726 | 0.812111449 | -0.142134067 | 1.6907E-06 | Hypomethylated |
| cg22668906 | 11 | IGR | LINC02098(dist=68741),ETS1(dist=148579) | 0.754227456 | 0.640441409 | 1.177668161 | 0.113786048 | 6.32949E-04 | Hypermethylated |
| cg22380254 | 11 | IGR | LINC02098(dist=77233),ETS1(dist=140087) | 0.880111896 | 0.574166986 | 1.532850055 | 0.30594491 | 5.88071E-07 | Hypermethylated |
| cg19402503 | 11 | IGR | LINC02098(dist=100240),ETS1(dist=117080) | 0.122148965 | 0.283867685 | 0.430302467 | -0.16171872 | 1.18622E-05 | Hypomethylated |
| cg00206381 | 11 | IGR | LINC02098(dist=152908),ETS1(dist=64412) | 0.210671922 | 0.315397083 | 0.667957739 | -0.104725161 | 3.32100E-05 | Hypomethylated |
| cg04372796 | 11 | TSS1500 | ETS1 | 0.481910027 | 0.595717772 | 0.808956941 | -0.113807745 | 6.60331E-04 | Hypomethylated |
| cg22966160 | 11 | TSS1500 | ETS1 | 0.536935818 | 0.657038503 | 0.817206018 | -0.120102685 | 1.53539E-04 | Hypomethylated |
| cg27625507 | 11 | 5'UTR | ETS1 | 0.643564925 | 0.753309383 | 0.854316884 | -0.109744458 | 9.18087E-06 | Hypomethylated |
| cg12116540 | 11 | 5'UTR | ETS1 | 0.348312087 | 0.528771745 | 0.658719175 | -0.180459657 | 4.60335E-05 | Hypomethylated |
| cg10290679 | 11 | 1stExon | ETS1 | 0.136139739 | 0.261914572 | 0.519786808 | -0.125774833 | 2.79669E-04 | Hypomethylated |
| cg17711101 | 11 | IGR | ETS1(dist=34375),LOC101929538(dist=59307) | 0.387765436 | 0.503551993 | 0.770060375 | -0.115786557 | 1.06573E-06 | Hypomethylated |
| cg26894820 | 12 | 1stExon | LUM | 0.675208914 | 0.492005948 | 1.372359252 | 0.183202967 | 3.6489E-06 | Hypermethylated |
| cg23455212 | 12 | 1stExon | LUM(dist=146) | 0.710439044 | 0.480550461 | 1.478385938 | 0.229888583 | 7.10052E-06 | Hypermethylated |
| cg13716827 | 15 | IGR | C15orf54(dist=13063),THBS1(dist=313174) | 0.077411978 | 0.259595379 | 0.298202451 | -0.1821834 | 9.17640E-06 | Hypomethylated |
| cg09604788 | 15 | IGR | C15orf54(dist=19554),THBS1(dist=306683) | 0.708516444 | 0.582153517 | 1.217061176 | 0.126362927 | 8.85310E-05 | Hypermethylated |
| cg25641635 | 15 | IGR | C15orf54(dist=156990),THBS1(dist=169247) | 0.759839382 | 0.902911596 | 0.841543497 | -0.143072214 | 4.51253E-05 | Hypomethylated |
| cg14793944 | 15 | IGR | C15orf54(dist=173112),THBS1(dist=153125) | 0.540690459 | 0.651666427 | 0.829704335 | -0.110975968 | 2.27471E-06 | Hypomethylated |
| cg06718604 | 15 | IGR | C15orf54(dist=178859),THBS1(dist=147378) | 0.409614397 | 0.529037482 | 0.774263471 | -0.119423085 | 6.68366E-05 | Hypomethylated |
| cg25959516 | 15 | IGR | C15orf54(dist=219922),THBS1(dist=106315) | 0.378741458 | 0.505949322 | 0.748575878 | -0.127207864 | 8.36172E-05 | Hypomethylated |
| cg04300701 | 15 | Body | THBS1 | 0.803597134 | 0.913517892 | 0.879673119 | -0.109920758 | 1.87484E-04 | Hypomethylated |
| cg11776255 | 17 | IGR | LINC01989(dist=37665),CCL2(dist=33988) | 0.442719748 | 0.684786518 | 0.646507687 | -0.24206677 | 4.29052E-06 | Hypomethylated |
| cg01987353 | 17 | IGR | LINC01989(dist=58989),CCL2(dist=12664) | 0.701020871 | 0.905966583 | 0.773782261 | -0.204945712 | 2.48916E-06 | Hypomethylated |
| cg07949722 | 17 | IGR | LINC01989(dist=66019),CCL2(dist=5634) | 0.33488747 | 0.440825566 | 0.759682504 | -0.105938096 | 4.53353E-05 | Hypomethylated |
| cg12698626 | 17 | TSS1500 | CCL2(dist=838) | 0.173944723 | 0.335146102 | 0.519011625 | -0.161201379 | 2.52875E-06 | Hypomethylated |
| cg17864156 | 17 | Body | CCL2 | 0.356516318 | 0.500581643 | 0.71220414 | -0.144065324 | 1.71884E-07 | Hypomethylated |
| cg14598213 | 17 | Body | LOC100288866 | 0.351602601 | 0.239444661 | 1.468408609 | 0.112157941 | 1.22854E-05 | Hypermethylated |
| cg11807280 | 2 | IGR | MEIS1-AS3 | 0.045053997 | 0.191077746 | 0.235788824 | -0.146023749 | 2.38002E-06 | Hypomethylated |
| cg10835286 | 2 | IGR | MEIS1-AS3 | 0.364428042 | 0.484444316 | 0.752259919 | -0.120016274 | 3.98987E-06 | Hypomethylated |
| cg23279522 | 2 | IGR | MEIS1-AS3 | 0.208848427 | 0.398866632 | 0.523604658 | -0.190018206 | 2.3398E-06 | Hypomethylated |
| cg22490405 | 2 | Body | MEIS1 | 0.702439744 | 0.371027911 | 1.893226152 | 0.331411833 | 1.20636E-06 | Hypermethylated |
| cg05091060 | 2 | Body | MEIS1 | 0.865759094 | 0.596777085 | 1.450724425 | 0.268982009 | 4.24072E-06 | Hypermethylated |
| cg07915559 | 2 | Body | MEIS1 | 0.879694952 | 0.613139996 | 1.434737512 | 0.266554956 | 3.38866E-07 | Hypermethylated |
| cg12132133 | 2 | Body | MEIS1 | 0.788479142 | 0.450191527 | 1.751430436 | 0.338287615 | 4.7752E-08 | Hypermethylated |
| cg20774834 | 2 | Body | MEIS1 | 0.837759289 | 0.541606088 | 1.546805522 | 0.2961532 | 2.44206E-08 | Hypermethylated |
| cg18329218 | 2 | Body | MEIS1 | 0.782883471 | 0.47245158 | 1.657066045 | 0.310431891 | 1.17066E-07 | Hypermethylated |
| cg07809589 | 2 | Body | MEIS1 | 0.897006186 | 0.542004045 | 1.654980612 | 0.355002141 | 9.09446E-07 | Hypermethylated |
| cg16927242 | 2 | Body | MEIS1 | 0.831376175 | 0.4625168 | 1.797504817 | 0.368859375 | 1.0233E-07 | Hypermethylated |
| cg05496203 | 2 | Body | MEIS1 | 0.812881574 | 0.517681199 | 1.570235843 | 0.295200375 | 1.18581E-07 | Hypermethylated |
| cg21306540 | 2 | Body | MEIS1 | 0.797034055 | 0.509041936 | 1.565753228 | 0.287992119 | 7.02411E-07 | Hypermethylated |
| cg06796713 | 2 | Body | MEIS1 | 0.830545306 | 0.529033367 | 1.569929908 | 0.301511938 | 9.88916E-09 | Hypermethylated |
| cg23398076 | 2 | Body | MEIS1 | 0.903929728 | 0.664107409 | 1.361119776 | 0.239822319 | 3.38824E-07 | Hypermethylated |
| cg22982573 | 2 | Body | MEIS1 | 0.880702073 | 0.570265601 | 1.544371732 | 0.310436473 | 9.81798E-07 | Hypermethylated |
| cg00995986 | 2 | Body | MEIS1 | 0.949264396 | 0.686396957 | 1.38296708 | 0.262867438 | 5.87182E-07 | Hypermethylated |
| cg16758314 | 2 | Body | MEIS1-AS2 | 0.890523394 | 0.70266432 | 1.267352516 | 0.187859074 | 2.64328E-05 | Hypermethylated |
| cg03589296 | 2 | Body | MEIS1-AS2 | 0.654685342 | 0.515576251 | 1.269812837 | 0.139109091 | 6.60339E-05 | Hypermethylated |
| cg06833110 | 2 | Body | MEIS1 | 0.092818766 | 0.215308853 | 0.43109591 | -0.122490087 | 8.85299E-05 | Hypomethylated |
| cg02551743 | 2 | Body | MEIS1 | 0.318998702 | 0.468307944 | 0.681172946 | -0.149309242 | 1.82113E-05 | Hypomethylated |
| cg04751149 | 2 | Body | MEIS1 | 0.290202842 | 0.393458943 | 0.737568296 | -0.103256101 | 1.77051E-05 | Hypomethylated |
| cg21715346 | 2 | Body | MEIS1 | 0.344291765 | 0.466917099 | 0.737372364 | -0.122625334 | 1.33211E-04 | Hypomethylated |
| cg08238215 | 2 | Body | MEIS1 | 0.76284388 | 0.865531892 | 0.881358489 | -0.102688012 | 9.08533E-04 | Hypomethylated |
| cg08791563 | 2 | Body | MEIS1 | 0.575527238 | 0.312725967 | 1.840356408 | 0.262801271 | 1.27231E-07 | Hypermethylated |
| cg27591920 | 2 | Body | BCL2L11 | 0.322995033 | 0.493714782 | 0.65421382 | -0.170719748 | 2.7775E-07 | Hypomethylated |
| cg24336374 | 21 | 3'UTR | RUNX1 | 0.703305194 | 0.80609573 | 0.872483463 | -0.102790536 | 4.10285E-05 | Hypomethylated |
| cg06862831 | 21 | 3'UTR | RUNX1 | 0.674696629 | 0.820322389 | 0.822477403 | -0.145625761 | 9.9922E-06 | Hypomethylated |
| cg02233071 | 21 | Body | RUNX1 | 0.305635672 | 0.406148024 | 0.752522859 | -0.100512352 | 3.09366E-05 | Hypomethylated |
| cg01018731 | 21 | Body | RUNX1 | 0.921874637 | 0.803359887 | 1.147523858 | 0.11851475 | 4.60940E-05 | Hypermethylated |
| cg27374486 | 21 | Body | RUNX1 | 0.854697314 | 0.747135905 | 1.143964984 | 0.107561409 | 3.86185E-04 | Hypermethylated |
| cg01854462 | 21 | IGR | RUNX1(dist=154539),LOC100506403(dist=168671) | 0.375059753 | 0.146102708 | 2.567096515 | 0.228957045 | 8.33555E-07 | Hypermethylated |
| cg09200260 | 21 | IGR | RUNX1(dist=155944),LOC100506403(dist=167266) | 0.691379847 | 0.264737502 | 2.611567462 | 0.426642345 | 9.0589E-09 | Hypermethylated |
| cg12480115 | 21 | IGR | RUNX1(dist=156008),LOC100506403(dist=167202) | 0.56135512 | 0.153597828 | 3.654707406 | 0.407757292 | 2.24676E-07 | Hypermethylated |
| cg21172011 | 21 | IGR | RUNX1(dist=156043),LOC100506403(dist=167167) | 0.482784949 | 0.142248477 | 3.393955145 | 0.340536473 | 2.12717E-07 | Hypermethylated |
| cg10719727 | 21 | IGR | RUNX1(dist=156248),LOC100506403(dist=166962) | 0.342001323 | 0.076421749 | 4.475183153 | 0.265579574 | 5.63207E-09 | Hypermethylated |
| cg15664596 | 21 | IGR | RUNX1(dist=207292),LOC100506403(dist=115918) | 0.200252159 | 0.076917734 | 2.603458907 | 0.123334426 | 5.45208E-06 | Hypermethylated |
| cg18099556 | 21 | IGR | RUNX1(dist=267839),LOC100506403(dist=55371) | 0.485413774 | 0.280738423 | 1.7290607 | 0.204675351 | 6.27433E-07 | Hypermethylated |
| cg02104409 | 22 | Body | SYN3,TIMP3 | 0.352742253 | 0.529053741 | 0.666741817 | -0.176311488 | 2.15711E-07 | Hypomethylated |
| cg02752076 | 3 | IGR | GPR149(dist=474271),MME(dist=119663) | 0.496574464 | 0.617019847 | 0.804794961 | -0.120445384 | 7.27903E-05 | Hypomethylated |
| cg18654931 | 3 | Body | MME | 0.705774956 | 0.829474677 | 0.850869803 | -0.123699722 | 0.001379271 | Hypomethylated |
| cg10314778 | 3 | IGR | MME(dist=708) | 0.332229633 | 0.226738955 | 1.465251673 | 0.105490678 | 7.78297E-05 | Hypermethylated |
| cg10969292 | 3 | IGR | MME(dist=6608),LINC01487(dist=50608) | 0.582254929 | 0.336062838 | 1.73257755 | 0.246192091 | 1.16756E-05 | Hypermethylated |
| cg14766448 | 3 | IGR | GHSR(dist=5261),TNFSF10(dist=51791) | 0.797247924 | 0.695444167 | 1.146386672 | 0.101803757 | 6.06434E-05 | Hypermethylated |
| cg11979312 | 3 | TSS1500 | TNFSF10(dist=1493),LINC02068(dist=35978) | 0.678650637 | 0.553342286 | 1.226457212 | 0.125308351 | 9.58832E-05 | Hypermethylated |
| cg08033580 | 5 | IGR | LOC101927190(dist=62406),SEMA6A(dist=6578) | 0.205560472 | 0.319279658 | 0.64382577 | -0.113719186 | 1.19513E-06 | Hypomethylated |
| cg13105555 | 5 | Body | SEMA6A-AS1 | 0.460905217 | 0.742431617 | 0.620804942 | -0.2815264 | 2.26715E-04 | Hypomethylated |
| cg11586894 | 5 | Body | SEMA6A | 0.667222608 | 0.808964504 | 0.824786014 | -0.141741895 | 0.033825094 | Hypomethylated |
| cg24941656 | 5 | Body | SEMA6A | 0.866387957 | 0.738772513 | 1.172739838 | 0.127615444 | 2.95777E-04 | Hypermethylated |
| cg25066845 | 5 | IGR | SEMA6A-AS2(dist=2980),LINC02214(dist=129122) | 0.519786538 | 0.694646656 | 0.748274729 | -0.174860118 | 4.40283E-08 | Hypomethylated |
| cg15747177 | 5 | TSS1500 | SQSTM1 | 0.411806006 | 0.515127769 | 0.799424979 | -0.103321763 | 1.42808E-04 | Hypomethylated |
| cg01607006 | 6 | IGR | MRPS18A(dist=53876),VEGFA(dist=28521) | 0.528048878 | 0.728986196 | 0.724360599 | -0.200937318 | 5.39028E-07 | Hypomethylated |
| cg18022921 | 6 | TSS1500 | VEGFA(dist=973) | 0.67091477 | 0.773081923 | 0.867844338 | -0.102167153 | 0.001981223 | Hypomethylated |
| cg23813001 | 7 | IGR | MAGI2-AS3(dist=335000),GNAI1(dist=328623) | 0.852944137 | 0.716840453 | 1.189866075 | 0.136103683 | 0.0000559 | Hypermethylated |
| cg06266516 | 7 | IGR | MAGI2-AS3(dist=508575),GNAI1(dist=155048) | 0.791210271 | 0.670170411 | 1.18061057 | 0.12103986 | 6.51831E-04 | Hypermethylated |
| cg24445278 | 7 | IGR | MAGI2-AS3(dist=513227),GNAI1(dist=150396) | 0.283754426 | 0.407223685 | 0.696802362 | -0.123469259 | 1.39419E-04 | Hypomethylated |
| cg14974807 | 7 | IGR | MAGI2-AS3(dist=639289),GNAI1(dist=24334) | 0.391894856 | 0.572336607 | 0.684727922 | -0.180441751 | 5.26108E-07 | Hypomethylated |
| cg15066837 | 7 | Body | GNAI1 | 0.359993748 | 0.472067882 | 0.762588944 | -0.112074134 | 1.65646E-06 | Hypomethylated |
| cg25418001 | 7 | Body | GNAI1 | 0.550858419 | 0.435499717 | 1.264888122 | 0.115358702 | 3.20868E-04 | Hypermethylated |
| cg12636421 | 7 | IGR | GNAI1(dist=143299),LOC101927269(dist=2038) | 0.149458474 | 0.045345991 | 3.29595782 | 0.104112483 | 1.56255E-05 | Hypermethylated |
| cg27287833 | 7 | IGR | BET1(dist=56647),COL1A2-AS1(dist=306210) | 0.392027985 | 0.514418405 | 0.762080014 | -0.12239042 | 5.87638E-04 | Hypomethylated |
| cg06748066 | 7 | IGR | BET1(dist=153722),COL1A2-AS1(dist=209135) | 0.367538903 | 0.515926409 | 0.712386295 | -0.148387506 | 0.005391723 | Hypomethylated |
| cg16520685 | 7 | IGR | BET1(dist=351222),COL1A2-AS1(dist=11635) | 0.905992836 | 0.731554052 | 1.238449618 | 0.174438784 | 2.13975E-06 | Hypermethylated |
| cg06261354 | 7 | TSS1500 | COL1A2-AS1(dist=1178),COL1A2(dist=1525) | 0.478423182 | 0.611443554 | 0.782448647 | -0.133020373 | 1.23881E-04 | Hypomethylated |
| cg10258721 | 7 | TSS1500 | COL1A2(dist=765) | 0.764113852 | 0.917526655 | 0.832797442 | -0.153412804 | 1.26712E-05 | Hypomethylated |
| cg23348014 | 7 | TSS1500 | COL1A2(dist=724) | 0.259374743 | 0.394621803 | 0.657274235 | -0.135247059 | 1.97764E-06 | Hypomethylated |
| cg08695855 | 7 | TSS200 | COL1A2(dist=464) | 0.297940052 | 0.413905596 | 0.719826101 | -0.115965545 | 1.91834E-04 | Hypomethylated |
| cg10368049 | 7 | TSS200 | COL1A2(dist=411) | 0.558901113 | 0.750689878 | 0.74451665 | -0.191788765 | 1.42922E-07 | Hypomethylated |
| cg18511007 | 7 | TSS200 | COL1A2(dist=385) | 0.280211364 | 0.411958851 | 0.680192606 | -0.131747487 | 2.83339E-05 | Hypomethylated |
| cg26942275 | 7 | TSS200 | COL1A2(dist=382) | 0.302546215 | 0.422838787 | 0.715511973 | -0.120292573 | 3.87190E-05 | Hypomethylated |
| cg09146903 | 7 | TSS200 | COL1A2(dist=338) | 0.235035857 | 0.358208687 | 0.656142257 | -0.123172831 | 1.08625E-06 | Hypomethylated |
| cg25300386 | 7 | 1stExon | COL1A2(dist=219) | 0.138200327 | 0.289638028 | 0.47714842 | -0.1514377 | 7.31666E-06 | Hypomethylated |
| cg08526705 | 8 | Body | MYC | 0.516115408 | 0.616474936 | 0.837204203 | -0.100359529 | 6.99169E-05 | Hypomethylated |
| cg17588094 | 8 | IGR | MYC(dist=73) | 0.174977936 | 0.28276331 | 0.618814143 | -0.107785375 | 1.69214E-04 | Hypomethylated |
| cg14752089 | 8 | IGR | MYC(dist=17845),PVT1(dist=33737) | 0.487751368 | 0.641527885 | 0.760296442 | -0.153776517 | 4.47905E-06 | Hypomethylated |
| cg04766289 | 8 | IGR | MYC(dist=33726),PVT1(dist=17856) | 0.787259542 | 0.684788335 | 1.149639242 | 0.102471208 | 6.89658E-04 | Hypermethylated |
| cg00163372 | 8 | Body | MYC | 0.498987 | 0.62051869 | 0.804144542 | -0.121531972 | 0.0000799 | Hypomethylated |
| cg06581465 | 9 | Body | GNAQ | 0.372840438 | 0.601173628 | 0.620187614 | -0.22833319 | 2.73098E-06 | Hypomethylated |
| cg00303252 | 9 | Body | GNAQ | 0.67671722 | 0.276634593 | 2.446249447 | 0.400082627 | 3.76058E-09 | Hypermethylated |
| cg25945990 | 9 | Body | GNAQ | 0.624334843 | 0.447466297 | 1.395266743 | 0.176868546 | 6.78554E-08 | Hypermethylated |
| cg17923271 | 9 | Body | GNAQ | 0.04677722 | 0.163598517 | 0.285926922 | -0.116821297 | 1.90795E-05 | Hypomethylated |
| cg05346624 | 9 | IGR | GNAQ(dist=132160),CEP78(dist=72104) | 0.694617182 | 0.840763802 | 0.826173987 | -0.14614662 | 0.001250362 | Hypomethylated |
| cg25346029 | 9 | IGR | GNAQ(dist=175937),CEP78(dist=28327) | 0.365936582 | 0.47935315 | 0.763396635 | -0.113416568 | 0.003478234 | Hypomethylated |
| cg21238276 | 9 | TSS1500 | GNAQ(dist=203073),CEP78(dist=1191) | 0.796743512 | 0.626454581 | 1.271829652 | 0.170288931 | 1.78253E-06 | Hypermethylated |

# Supplementary Table 2 Differentially expressed methylation sites

| **Target** | **Position** | **P-value (Ttest)** | **OGD_Mean** | **OGD_StdDev** | **NC_Mean** | **NC_StdDev** |
| --- | --- | --- | --- | --- | --- | --- |
| BCL2L11_2_1_ | 53 | 0.026476613 | 0.019090709 | 0.002541356 | 0.025843647 | 0.001314497 |
| BCL2L11_2_2_ | 74 | 0.045636821 | 0.011514552 | 0.002835262 | 0.005445865 | 0.001505914 |
| BCL2L11_2_2_ | 121 | 0.030822393 | 0.012358617 | 0.000980206 | 0.015641765 | 0.001343747 |
| BDNF_1_ | 193 | 0.025923223 | 0.007072965 | 0.002080444 | 0.016506123 | 0.003595044 |
| BDNF_2_ | 30 | 0.028979373 | 0.008627525 | 0.001186743 | 0.016612208 | 0.002941152 |
| BDNF_2_ | 42 | 0.033513044 | 0.042712141 | 0.00420102 | 0.031797712 | 0.001565138 |
| BDNF_4_ | 58 | 0.042106327 | 0.010916304 | 0.001886216 | 0.006185047 | 0.002034413 |
| BDNF_4_ | 186 | 0.015284372 | 0.006369256 | 0.001978965 | 0.013491374 | 0.000754055 |
| CD44_2_ | 176 | 0.01990883 | 0.022109898 | 0.003585634 | 0.011804089 | 0.002115165 |
| COL1A2_ | 58 | 0.041094526 | 0.04612667 | 0.014410152 | 0.079818528 | 0.005827624 |
| ETS1_2_NEW | 176 | 0.013407696 | 0.006403545 | 0.001201912 | 0.012550717 | 0.001893358 |
| GNAI1_2_ | 96 | 0.005685328 | 0.021642672 | 0.001737146 | 0.029929974 | 0.00196783 |
| MAP3K5_1_2_ | 187 | 0.012773317 | 0.003181733 | 0.001157755 | 0.012477084 | 0.002553821 |
| MEIS1_4_ | 39 | 0.022452771 | 0.004570944 | 0.001011719 | 0.007862051 | 0.001183052 |
| MEIS1_4_ | 43 | 0.046878057 | 0.012842631 | 0.000931826 | 0.014881236 | 0.000807914 |
| MME_ | 122 | 0.019414817 | 0.00772543 | 0.000924282 | 0.003499938 | 0.001471556 |
| MME_ | 152 | 0.038957947 | 0.010028218 | 0.000898712 | 0.006714866 | 0.001474723 |
| MYC_3_ | 45 | 0.040536344 | 0.010595016 | 0.001427089 | 0.007183698 | 0.000530718 |
| MYC_3_ | 61 | 0.010468112 | 0.011397549 | 0.000703688 | 0.008944947 | 0.000473837 |
| MYC_5_ | 179 | 0.022859634 | 0.008753087 | 0.001480336 | 0.004730121 | 0.001157477 |
| NGFR_1_ | 157 | 0.021742923 | 0.016788565 | 0.000635106 | 0.005836558 | 0.003084177 |
| NGFR_1_ | 207 | 0.018846634 | 0.009920431 | 0.003427171 | 0.020466184 | 0.00333943 |
| NGFR_2_1_ | 28 | 0.048932174 | 0.016339869 | 0.014975738 | 0.067180425 | 0.024669698 |
| RUNX1_1_ | 87 | 0.031349285 | 0.00709888 | 0.000263458 | 0.010591187 | 0.001186248 |
| RUNX1_4_1_ | 135 | 0.033482149 | 0.012819081 | 0.003916562 | 0.003474123 | 0.002285522 |
| RUNX1_4_1_ | 203 | 0.043864241 | 0.009438097 | 0.001354104 | 0.006090936 | 0.000326642 |
| RUNX1_5_NEW | 86 | 0.000604337 | 0.012850198 | 0.000999366 | 0.004870848 | 0.000713269 |
| RUNX3_2_ | 54 | 0.016555614 | 0.215571264 | 0.014048044 | 0.259682227 | 0.007486907 |
| RUNX3_2_ | 186 | 0.005003593 | 0.033695675 | 0.001311215 | 0.047152613 | 0.002694917 |
| RUNX3_4_ | 181 | 0.022913974 | 0.021242149 | 0.002653376 | 0.01378477 | 0.001363578 |
| SEMA6A_2_ | 60 | 0.011638919 | 0.011500254 | 0.001290234 | 0.006939602 | 0.000704648 |
| SEMA6A_3_ | 74 | 0.028342384 | 0.007885152 | 0.001980591 | 0.012925364 | 0.00113108 |
| SEMA6A_3_ | 164 | 0.041922783 | 0.003881493 | 9.45348E-05 | 0.009059751 | 0.001903853 |
| SEMA6A_5_NEW | 125 | 0.033366429 | 0.010405644 | 0.002018551 | 0.005282865 | 0.00191441 |
| SQSTM1_1_ | 92 | 0.015207741 | 0.022718019 | 0.006451152 | 0.002824859 | 0.004892799 |
| SQSTM1_3_ | 129 | 0.017648611 | 0.968014245 | 0.004404187 | 0.954938798 | 0.002749405 |
| TIMP3_ | 165 | 0.04656394 | 0.010820466 | 0.00139611 | 0.019446947 | 0.003826664 |
| TNFRSF9_ | 146 | 0.048443709 | 0.007574861 | 0.004603173 | 0.017241274 | 0.003541989 |
| TNFRSF9_ | 168 | 0.00648791 | 0.027080872 | 0.004002145 | 0.009505632 | 0.0042418 |
| VEGFA_3_ | 34 | 0.021571477 | 0.003081039 | 0.000238255 | 0.006481307 | 0.000980192 |
| VEGFA_4_NEW | 57 | 0.016643755 | 0.00847953 | 0.000935635 | 0.011405207 | 0.000865092 |
| VEGFA_4_NEW | 174 | 0.039691194 | 0.011129889 | 0.00267554 | 0.005087728 | 0.002083744 |

# Supplementary Table 3 Significantly Different in Abundance of Methylation Haplotype

| **Target** | **Haplotype** | **P-value(Ttest)** | **OGD_Mean** | **OGD_StdDev** | **NC_Mean** | **NC_StdDev** |
| --- | --- | --- | --- | --- | --- | --- |
| BCL2L11_2_1_ | tttcttttttttttt | 0.027136773 | 0.013811202 | 0.002101138 | 0.019896013 | 0.000728568 |
| BDNF_1_ | ttttttttttttttttttt | 0.033488442 | 0.872671804 | 0.012617378 | 0.840673243 | 0.005256486 |
| BDNF_1_ | ttttttttttttctttttt | 0.012942043 | 0.004862965 | 0.002156505 | 0.015029099 | 0.003128693 |
| BDNF_2_ | ttttttttttttttc | 0.035047374 | 0.021424432 | 0.003844532 | 0.012039735 | 0.001755097 |
| BDNF_4_ | tttttttttttttttttttct | 0.012026774 | 0.00470365 | 0.001372005 | 0.01179578 | 8.06024E-05 |
| CD44_1_ | ttttttttttctt | 0.033264901 | 0.012846791 | 0.00155323 | 0.008718704 | 0.000528361 |
| ETS1_1_ | ttcttttttttt | 0.022461882 | 0.009413386 | 0.001288918 | 0.012973665 | 0.001070628 |
| MYC_1_ | ttttttttttttt | 0.022230347 | 0.891349119 | 0.005878888 | 0.909562111 | 0.006371118 |
| NGFR_1_ | ttttttttctttttttttttttttttt | 0.011560462 | 0.019780179 | 0.002890686 | 0.035332043 | 0.004560659 |
| NGFR_1_ | tttttttttttttttttttttttttct | 0.040188285 | 0.005086968 | 0.004412171 | 0.016817162 | 0.005080864 |
| SEMA6A_2_ | ttttttctttttttt | 0.038345663 | 0.010408834 | 0.001669986 | 0.005780305 | 0.000241683 |
| SEMA6A_3_ | ttttttttttttttttttttttt | 0.048134031 | 0.809972743 | 0.011439584 | 0.781288053 | 0.013252415 |
| SQSTM1_1_ | ttttttttctttttttt | 0.019244979 | 0.020447886 | 0.004985487 | 0 | 0 |
| SQSTM1_2_NEW | tttttttttttttttttttt | 0.014563733 | 0.83669414 | 0.002444616 | 0.859546383 | 0.006272205 |
| TNFRSF9_ | tttttttttcttttttttttt | 0.018280896 | 0.016592632 | 0.004198236 | 0.004328259 | 0.00270705 |
